# An effective simulation- and measurement-based workflow for enhanced diagnostics in rhinology

**DOI:** 10.1007/s11517-021-02446-3

**Published:** 2021-12-23

**Authors:** Moritz Waldmann, Alice Grosch, Christian Witzler, Matthias Lehner, Odo Benda, Walter Koch, Klaus Vogt, Christopher Kohn, Wolfgang Schröder, Jens Henrik Göbbert, Andreas Lintermann

**Affiliations:** 1 Institute of Aerodynamics and Chair of Fluid Mechanics, RWTH Aachen University, Wüllnerstr. 5a, 52062 Aachen, Germany; 2 Jülich Supercomputing Centre, Forschungszentrum Jülich GmbH, Wilhelm-Johnen-Straße, 52425 Jülich, Germany; 3 Jülich Aachen Research Alliance Center for Simulation and Data Science (JARA-CSD), RWTH Aachen University and Forschungszentrum Jülich GmbH, Kopernikusstr. 6, 52074 Aachen, Germany; 4 Angewandte Informationstechnik Forschungsgesellschaft mbH, Klosterwiesgasse 32/1, 8010 Graz, Austria; 5 Sutter Medizintechnik GmbH, Tullastraße 87, 79108 Freiburg, Germany; 6 Med Contact GmbH, Kornbühlstr. 100-102, 72393 Salmendingen, Germany

**Keywords:** Rhinology, High-performance computing, Computational fluid dynamics, Machine learning

## Abstract

Physics-based analyses have the potential to consolidate and substantiate medical diagnoses in rhinology. Such methods are frequently subject to intense investigations in research. However, they are not used in clinical applications, yet. One issue preventing their direct integration is that these methods are commonly developed as isolated solutions which do not consider the whole chain of data processing from initial medical to higher valued data. This manuscript presents a workflow that incorporates the whole data processing pipeline based on a Jupyter environment. Therefore, medical image data are fully automatically pre-processed by machine learning algorithms. The resulting geometries employed for the simulations on high-performance computing systems reach an accuracy of up to 99.5% compared to manually segmented geometries. Additionally, the user is enabled to upload and visualize 4-phase rhinomanometry data. Subsequent analysis and visualization of the simulation outcome extend the results of standardized diagnostic methods by a physically sound interpretation. Along with a detailed presentation of the methodologies, the capabilities of the workflow are demonstrated by evaluating an exemplary medical case. The pipeline output is compared to 4-phase rhinomanometry data. The comparison underlines the functionality of the pipeline. However, it also illustrates the influence of mucosa swelling on the simulation.

**Graphical Abstract:**
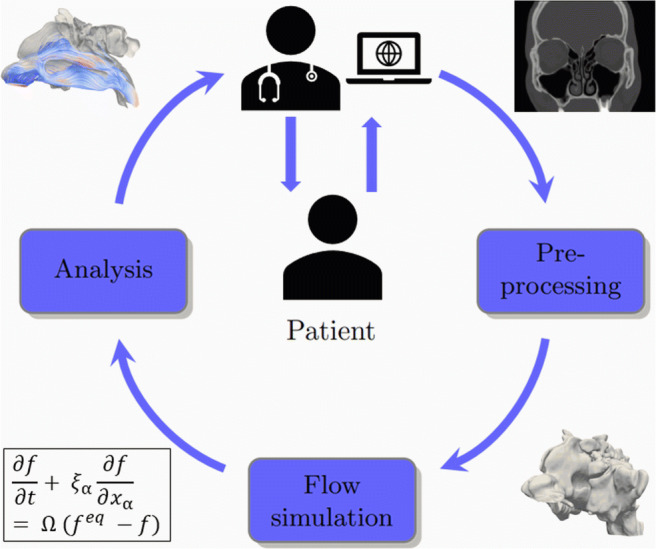
Workflow for enhanced diagnostics in rhinology.

Workflow for enhanced diagnostics in rhinology.

## Introduction

The nasal cavity is one of the most important organs of the human body. Its various functionalities are essential for the well-being of the individual person. The nasal cavity is responsible for the sense of smell, supports degustation, and filters, tempers, and moistens the inhaled air to provide optimal conditions for the lung. Diseases of the nasal cavity like chronic rhinosinusitis, septal deviation, or nasal polyps may lead to restrictions or complete loss of these functionalities [11, 12]. A decreased respiratory capability, the development of irritations and inflammations, and lung diseases can be the consequences. A meaningful and physics-based diagnosis is hence essential to adequately understand the functional efficiency of the nasal cavity and the impact of different pathologies.

Nowadays, the diagnostic quality is primarily based on the experience of the corresponding physicians. Morphological diagnoses frequently employ methods of medical imaging such as computed tomography (CT) or magnetic resonance imaging (MRI), and nasal endoscopy [44]. Such methods, however, do not cover the fluid mechanics of respiration. A physically correct analysis of the nasal airflow has to consider at minimum two parameters: (i) the mass flux and (ii) the differential pressure between the nares and nasopharynx.

Before digitized measurement techniques employed personal computers, rhinomanometry had been the gold standard to analyze physical respiratory quantities. In this method, both parameters (i) and (ii) are read from *xy*-plots. Therefore, it is necessary to set a reference point at 150 *Pa*, which is standardized through many years by the ISCOANA (International Standardization Committee on the Objective Assessment of the Upper Airway) [9, 10]. The analysis of the entire respiration cycle and the subsequent calculation of the resistance by root-mean-square (RMS) values of the pressure and mass flux, and the logarithmic transformation of these values lead to the so called 4-phase rhinomanometry (4-PR) [46]. This advanced method of resistance measurement is the only method correlating subjective sensing of nasal obstruction. The plots generated by this method allow the estimate of the influence of elastic compartments in the vicinity of the nasal entrance at inspiration. In multicentric studies [47, 48], a classification of the obstruction is obtained from 36,500 measurements for a single nasal side and from 10,000 for the total nose. The measurements were performed on patients in the age of 20 to 85 years. From the corresponding findings, five categories that classify nasal cavities by their resistance are derived. They can be used by clinicians to support surgery decisions impacting the shape of nasal cavities. A classification for the age between 6 and 19 years can be added using a correction following the statistically relevant investigations in growing noses [40]. This advanced method of rhinomanometry is recommended as the new standard in [45, 48]. 4-PR is a low-cost procedure, which can be carried out in any clinical unit. The results underline that there is a necessity to determine the impact of the morphology of the nasal cavity on the corresponding respiration physics. In contrast, the frequently used acoustic rhinometry does not analyze the functional obstruction. It only measures the cross-sectional area in the anterior thirds of the nasal cavity. Furthermore, the cheap and widely used peak nasal inspiration flow (PNIF) [53] method does not consider the motility of the nasal valve.

Multiple studies already employ computational fluid dynamics (CFD) simulations to assess the physics of respiration [2, 5–7, 14, 16, 28–32, 35, 36, 50]. However, none of them has made it into clinical application so far. Most of the studies solve the Reynolds-Averaged Navier-Stokes (RANS) equations, i.e., turbulent structures are not fully resolved, but are modeled using different kinds of turbulence models. Models such as the *k*-*𝜖* [8] or the *k*-*ω* model [51] have been developed for specific flow problems. They are able to produce accurate results for their corresponding specific kind of problem. For others, they may result in less convincing results, depending on the specifics of the model, which need to be tuned for each individual simulation case. Especially for complex fluid mechanical problems in intricate geometries such as the flow in the human respiratory system, their results may fundamentally differ from reality. In contrast, large-eddy simulations (LES) [2, 6, 7] and direct numerical simulations (DNS) [14, 28–32, 35, 36, 50] lead to more confident results. These methods promise computer-aided flow predictions for individual patients and allow to detect anatomical locations of pathologies. Despite their computational cost, such detailed simulations allow the necessary accuracy for surgery planning. Utilizing high-performance computing (HPC) systems reduces the time-to-solution required for LES and DNS. In the present study, a thermal lattice-Boltzmann (TLB) method as part of the multiphysics software m-AIA (multiphysics Aerodynamisches Institut Aachen), formerly known as Zonal Flow Solver (ZFS), framework [33] is employed for the simulation of the respiratory flow and the temperature distribution. No model of any filtered scales is considered. The LB method allows for efficient parallelization and is hence well suited for massively parallel computations on HPC systems.

A critical prerequisite to perform highly accurate CFD simulations of nasal airflow is the accurate reconstruction of nasal geometries from medical imaging data. Anatomically plausible 3D polygonal mesh models, e.g., in standard tessellation language (STL), are the base for such simulations. Therefore, CT volume data sets need to be segmented in order to classify anatomical structures that are of interest for CFD purposes. That is, the nasal cavity, the paranasal sinuses, or the oral cavity need to be separated from bone, tissue, air inside the mastoid air cells, and air outside of the patient’s head. Manual or semi-automatic segmentation using software such as 3D Slicer (https://www.slicer.org) or Materialise (https://www.materialise.com) is tedious and requires experienced personnel. Using these software tools it can be time-consuming to obtain a high-quality air segmentation for an individual patient, at least if accurate segmentations of delicate structures like the thin meatus of the nose and the ethmoidal air cells are needed. Once an air segmentation has been obtained, it is laborious to separate, e.g., the nasal cavity from the paranasal sinuses and the air outside the head. Furthermore, the tremendous anatomic variability of the nasal cavity makes it difficult to use a simple rule-based approach to automatically obtain high-quality segmentations of CT volumes for different patients. This is further complicated by high noise, limited resolution, and suboptimal discriminability of different tissues on a per voxel basis of CT data. Obviously, these obstacles pose fundamental challenges to analyzing large amounts of patient data. Hence, it is mandatory to develop pipelines that allow the segmentation of medical image data and generate computational meshes in a completely automated manner while yielding morphologically accurate results rapidly.

In recent years, machine learning (ML) algorithms have outperformed many techniques previously considered state-of-the-art [49]. Convolutional neural networks (CNNs) nowadays beat classical approaches on a variety of computer vision tasks including object detection, face recognition, body part classification, and image segmentation. Litjens et al. [37] conduct a survey which covers over 300 publications from 2012 to 2017 on applications of deep learning (DL) techniques to analyze medical images. This survey reveals CNNs to be the most frequently used DL algorithms in medical image analysis in this time period. Moreover, CNNs are most commonly used for image segmentation, especially for MRI and CT images. Based on the work in [39] on optic disk and retinal vasculature segmentation in eye fundus photographs, CNNs are developed to obtain 2D segmentations of either air or bone in axial CT slices of the sinonasal cavities. In [39] highly accurate segmentations of delicate retinal blood vessels are obtained by a simple CNN architecture. The meatus of the nose, the ethmoid air cells and the bones surrounding the sinonasal cavities are similarly thin and intricate. Given the promising results obtained for CNN-based image segmentation in other areas of medical imaging, the current approach is also based on CNNs.

Workflows using HPC systems can become quite complex and setting them up requires a fair amount of expertise in computer science, software development, and HPC hardware. The command line is still the most widely used method to access and interact with HPC systems. Obviously, this leads to a high entry barrier. Furthermore, the computing resources are distributed piecewise and are frequently shared among many users. That is, compute nodes can be allocated for a certain time in batch mode and the corresponding compute jobs usually start at an undefined point in time in the future. This is due to the varying load of HPC systems, which is caused by user jobs allocating unforeseeable resources for an unforeseeable amount of time. This kind of scheduling is not known from computers at home, where different programs alternate very quickly and therefore, appear to run in parallel. Using such complex systems in clinical environments creates an extra hurdle for the users, is, however, necessary to yield sufficiently accurate simulation results.

To include all aforementioned necessary functionalities into a single framework, the workflow presented in this manuscript uses Jupyter (https://jupyter.org), which enables to use HPC systems through web browsers such as Firefox. The Jupyter extension voilà (https://voila.readthedocs.io) is used to create graphical interfaces that are similar in appearance to conventional websites. Considering numerical simulations, the workflow integrates three major steps: (i) ML-based methods for pre-processing medical image data are employed within Jupyter to prepare highly resolved simulations on HPC systems, (ii) the simulation itself is executed using the TLB method to simulate respiratory flows on HPC systems, and (iii) the evaluation of the results is performed. In addition to the first step, Jupyter takes care of efficiently post-processing and visualizing simulation data. The Jupyter environment with an in-situ coupling of the simulation with ParaView Catalyst (https://www.paraview.org/in-situ/) is furthermore used to monitor the simulation and to visualize simulation results live on a website. The workflow hence realizes a user interface that is intuitive and easy to use and yields a novel diagnostic tool for physicians.

In the following, the material and methods for establishing a full automatic simulation pipeline are discussed first. Subsequently, the results applying this pipeline to an exemplary medical case are presented. This is followed by a discussion. Finally, the work is summarized, some conclusions are drawn, and an outlook is given.

## Materials and methods

**Fig. 1:**
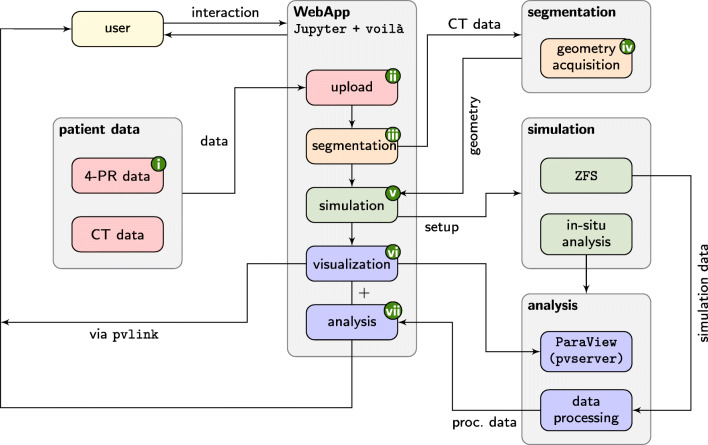
Jupyter-based framework for the simulation of respiratory flows and interactive supercomputing

To enrich standardized functional diagnosis with results from realistic, physics-based numerical simulations and from reliable experimental measurements, the whole simulation pipeline and 4-PR measurements are integrated into an easy extendable and accessible framework. The framework consists of the components outlined in Fig. 1. To provide a user-friendly access to the framework’s functionalities, a web-based Jupyter environment is developed that provides components to upload CT data sets, monitor HPC simulation, and visualize and analyze the simulation results. A typical case analysis may consist of the following steps:

(i) clinical 4-PR diagnostics evaluate the nasal respiratory capability in-vivo;
(ii) the user uploads a CT data set and the 4-PR results; the anonymization is performed on the client side;
(iii) ML algorithms segment the CT data set;
(iv) a three-dimensional geometrical representation of the nasal cavity is generated and prepared for a simulation;
(v) a stationary CFD simulation of the respiratory flow is performed on an HPC system;
(vi) at run time the results are processed and visualized;
(vii) the final results are presented to the user together with the uploaded 4-PR measurements;

In the following sections, the core components that constitute the pipeline from clinical diagnostics using 4-PR over CT data set upload and geometry acquisition to enhanced diagnostic results based on CFD are presented. That is, the 4-PR technology, the Jupyter environment, the methods for acquiring the geometry and the numerical simulations of the flow, in-situ visualization, and post-processing techniques are introduced.

### Four-phase rhinomanometry

**Fig. 2:**
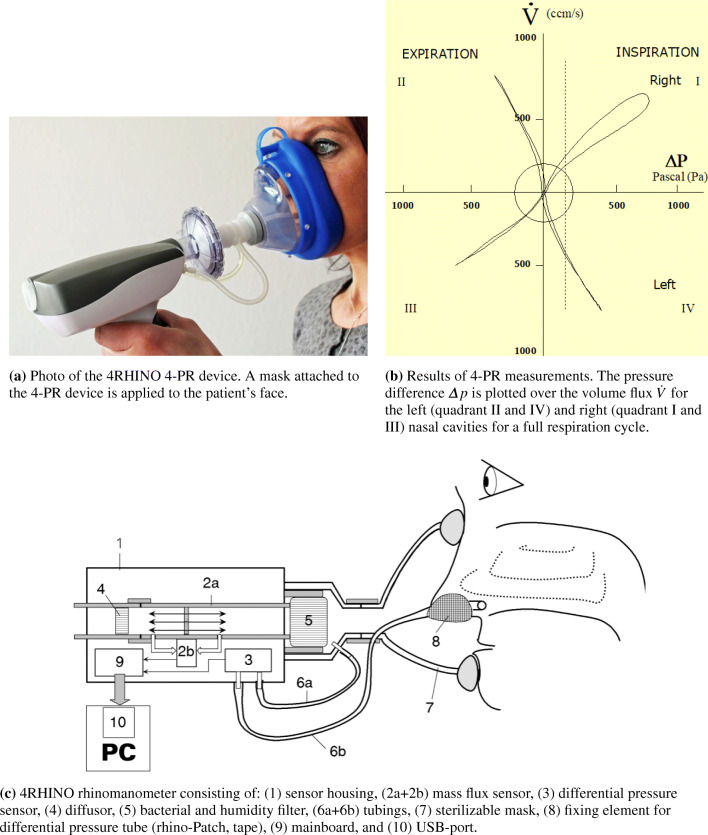
Photo of a 4-PR rhinomanometer device in application and the corresponding schematics. Furthermore, measurement results are shown. The energy supply of the measurement unit is provided via the USB-port

In clinical practice, rhinomanometry follows immediately the basic investigation of the patient, i.e., history review, inspection, and in some cases nasal endoscopy. Figure 2a shows a 4RHINO 4-PR device in application and Fig. 2b the results of a measurement. The details of a rhinomanometer are depicted in Fig. 2c. Unlike in anesthesiology or emergency, the airflow in 4-PR is determined by digitally calibrated mass flux sensors instead of pneumotachographs and digital pressure sensors. The measurements are usually carried out for breathing at rest. One nasal side is closed by a tape connecting the non-breathing side with a tube measuring the differential pressure Δ*p* between nasopharynx and a mask covering the nose and mouth of the patient. The method also allows the measurement of the total nasal resistance. A further method, which is in use worldwide, is the calculation of the total nasal resistance by the equation for parallel electric resistances. A classification of the resistances is published in [47]. Note that the accuracy of the estimation is presently under experimentally investigation. The influence of the mucosa on the resistance is determined by a decongestion test. Therefore, both nasal sides are measured before and 10 min after application of 0.1% xylometazoline nose drops. In case the influence of the body position is of interest, e.g., in sleep medicine, the measurements are repeated in the supine or lateral position. A recording of subjective complaints on a visual analogue scale (VAS) can be added to the measured results. Using the obstruction/resistance classes shown in Table 1, the findings from the 4-PR measurements can be used to quantitatively judge the grade of respiratory degradation and to decide if CFD analyses should be performed to obtain more detailed diagnostic information. The values within the clinical classification are calculated from the effective resistance

1 $$ R_{eff}=\frac{\Delta p_{eff}}{\dot{V}_{eff}}=\sqrt{\frac{1}{T}{\cdot{\int}_{0}^{T}}\frac{\Delta p}{\dot{V}}dt}, $$

**Table 1:** Resistance classification: one-sided logarithmic effective resistance $LR_{eff}^{1}$ and two-sided calculated logarithmic effective resistance $LR_{eff}^{2}$

Class	Obstruction/resistance	$LR_{eff}^{1}$ range	$LR_{eff}^{2}$ range
1	Very low	≤ 0.755	≤ 0.423
2	Low	0.760–0.960	0.575–0.703
3	Moderate	0.910–1.135	0.467–0.592
4	High	1.136–1.365	0.593–0.755
5	Very high	> 1.365	> 1.756

where *T* is the total measurement time over a single respiration cycle, *t* is the time, and $\dot {V}$ is the volume flux. Furthermore, the vertex resistances *VR* at inspiration, expiration, or for the complete respiration cycle is used. The vertex resistance is the resistance measured at the highest point of a respiration wave at quiet breathing, where a linear relation between mass flux $\dot {m}$ and pressure *p* exists [46]. Hence, it is defined completely different from the definition of the PNIF. The latter is measured at maximum inspiratory breathing and does not account for the movement of the nasal valve towards the septum.

A classification can then be derived by

2 $$ LR_{eff}^{1} = \log(10\cdot R_{eff})\stackrel{\scriptsize !}{\geq}0. $$

Using the bounds in Table 1 for $LR_{eff}^{1}$ and $LR_{eff}^{2}$, which are the measured one- and two-sided quantities, a classification is obtained. The latter is given by

3 $$ LR_{eff}^{2} = \log\left[10\cdot\frac{R_{eff}^{left} \cdot R_{eff}^{right}}{R_{eff}^{left} + R_{eff}^{right}}\right], $$

i.e., using the effective resistances on the *left* and *right* side. In any case, the results of the 4-PR measurements are fed into the data pipeline in the Jupyter environment. They can subsequently be visualized and be compared to the simulation outcome. For more details on the 4-PR measurement technique, the reader is referred to [45–48].

### Jupyter environment

The Jupyter environment provides a visible user interface. It is a browser-based tool that allows users to combine descriptions, source code, and results in Jupyter notebooks in a single place [18]. It enables to display results and how they have been generated in a documented and clear way, i.e., it advances reproducibility of (not only) scientific results. Using Jupyter’s voilà extension, experts and non-experts can use the same notebook. On the one hand, non-experts can be presented a normal website excluding source code for better interactivity. On the other hand, experts can easily make changes and analyze the results from individual points of view. The Jupyter notebooks are accessible through JupyterLab (https://jupyterlab.readthedocs.io). JupyterLab enables to use custom components and allows flexible arrangements of multiple different documents.

In this study, a JupyterLab extension is developed to copy CT data sets to the HPC systems at the Jülich Supercomputing Centre (JSC), Forschungszentrum Jülich. Therefore, a hash value of the image data is first generated using the message-digest algorithm 5 (MD5) [42]. The hash value is then transmitted and used to check for redundancy in JSC’s data base and for a later identification on the client side. All personal information is removed from the DICOM headers of the files to be transmitted. That is, a pseudo-anonymization takes place, where key pairs of the hash values and the patient information are stored in the hospital and only anonymized patient data arrives at JSC. The data is encrypted using AES-256 [13] and is then transmitted using an https-encrypted connection. The decryption key is stored separately from the data and only used when access is required. The system allows to add additional comments to the data set and to upload data acquired using 4-PR.

With the help of the extension pvlink, which is developed at JSC, visualization functionalities are integrated into JupyterLab. The extension makes use of ParaView (https://www.paraview.org) to interact with the various simulation in- and outputs in a web front-end. The data is rendered by ParaView on the server and presented in Jupyter. Using server side rendering on the HPC system enables the user to visualize data without being limited by their personal computer and circumvents the need to transfer all the data. This is realized by providing a web socket on the Jupyter server to the web client. The access is granted by a JavaScript, which is executed in the user’s browser. The script also registers the user’s interaction with the rendered picture and communicates corresponding operations such as zooming, panning, or rotating objects.

To allow for interaction with a running simulation, ParaView’s in-situ interface Catalyst is used in Jupyter. To make this interface work properly, it is necessary to replace some of ParaView’s graphical user interface (GUI) functionalities such that Jupyter is the interacting element. That is, functionalities are replaced by corresponding Jupyter scripts written in Python. This allows the connection of the simulation to the visualization, extraction of data, and update of the visualization.

### Geometry acquisition

For the purpose of segmenting incoming CT data sets automatically, a CNN is trained to generate 2D binary segmentations of air-filled regions in axial CT slices. Labeled data, generated by manually segmenting CT data sets, are provided as training sets to iteratively improve segmentations. Since the quality of the labeled data determines the accuracy of the outcome, the manual segmentation is performed by experts. The image segmentation task is implemented as a mathematical optimization problem, where a differentiable cost function is minimized by an iterative optimization algorithm. The cost function determines the difference between the predictions computed by the CNN and the labeled data, i.e., in the learning process it is the objective to minimize the cost function. Once trained, the CNN is able to generate segmentations for unseen CT data sets. In addition to simple air/non-air segmentations, CNNs are trained to identify different air-filled regions, i.e., the frontal, maxillary, and sphenoid sinuses on both sides of the body, the nasal cavity, and the air-filled region outside the head.

In the following, more details on the medical image data base, the manual labeling process, and the training of CNNs for binary segmentation and multi-class segmentation are provided.

#### Medical image data base

All CT slices have a full resolution of 512 *px*^2^. A single patient’s CT volume consists of roughly 200 axial slices.

The CNN is trained only for a slice thickness of 0.6 *mm*. As for all stochastic systems, imperfections can not be precluded. Positive as well as negative examples concerning the slice thicknesses in the range between 3.0 and 0.1 *mm* (CT vendor interpolated) were obtained. However, the impact on the likelihood of imperfections, e.g., failure rates of the geometric plausibility checks, was not investigated. The post-processing phase of the current implementation checks for size, position, and connectivity of the identified objects.

The first models are trained with 213 axial slices of a single patient. In this data set, no significant obstructions are noticeable and all sinuses are present. A 256 *px*^2^ CT slice subset, limiting the original data to the nasal cavity and sinuses, is used as input to a supervised learning process. This limitation removes parts of the image such as the mastoid air cells, which are very time-consuming and difficult to segment accurately, and restricts the analysis to the region of interest (ROI). From the 213 axial slices, 20 hand-selected images covering a wide range of anatomical regions are used as validation set. The remaining 193 images are used as training set. No test set is used for these initial experiments. To train a multi-class segmentation CNN with 9 classes, a data set consisting of 579 CT images from 3 different healthy patients is used. 511 of these are used as training set. Due to severe class imbalance, oversampling is used, which results in a final training set of 4,094 images. In the multi-class case, the ROI is chosen to have a size of 400 *px*^2^. As a pre-processing step, the intensity values are scaled to [0,1]. Table 2 summarizes the medical image data base used for training and validation.

**Table 2:** Medical image data base used for CNN training

Segmentation type	Patients	Slices	Train. set	Val. set	Test set	ROI [*px*^2^]
Binary (air or bone)	1	213	193	20	0	256
9 classes (unbalanced)	3	579	511	34	34	400
9 classes (balanced)	3	4,162	4,094	34	34	400

#### Manual labeling process

To generate initial training data, segmentations of a limited number of patients are created semi-automatically by using the Segment Editor module in the free open source software 3D Slicer. First, the threshold tool is used to assign voxels in a certain Hounsfield unit range to air and bone segments. Subsequently, a smoothing filter is applied followed by extensive manual editing. The tissues segment is then given by the remaining voxels, which belong neither to the air nor to the bone segments. For the multi-class segmentation individual labeling then is performed. To ensure high quality of the segmentations, they are checked for anatomic plausibility by an ENT surgeon specialized in sinus surgery.

#### CNNs for binary segmentation

**Fig. 3:**
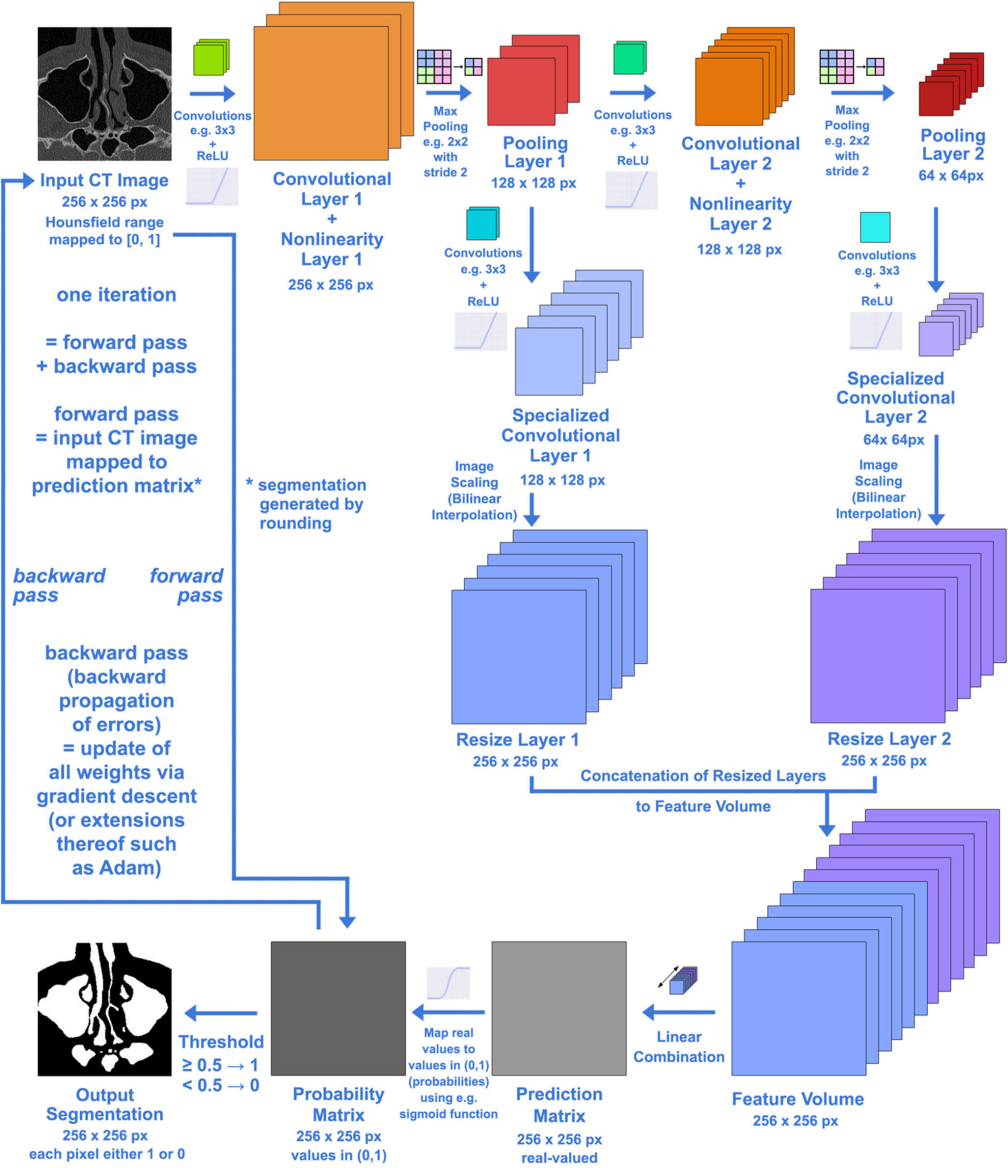
Basic binary segmentation CNN architecture for a 256 *px*^2^ ROI

Figure 3 shows the basic architecture of a binary segmentation CNN with 2 convolutional layers. The network weights are initialized randomly and drawn from a truncated normal distribution. For training, a batch size of *BS* = 1 is used. The optimization is performed with the Adam algorithm [24]. The CNN contains 1 to 4 convolutional layers and further specialized convolutional layers, all with a filter size of 3 × 3 with 5 to 20 filters per layer. A fixed learning rate of 0.01 is used. This set of hyperparameters leads to convergence, defined as a residual below 10^− 5^ for 30 consecutive iterations, after anywhere from 27 to 2,078 iterations.

The models are trained on a NVIDIA GTX 1080 GPU. The class-balanced cross entropy introduced by Xie et al. [52] is used as cost function. For a single pre-processed input image *X*_*n*_,*n* ∈{1,…,*N*}, the cost is given by

4 $$ \begin{array}{@{}rcl@{}} C_{n}(\boldsymbol{W}) &= & -\beta_{n} \sum\limits_{j \in Y_{n}^{+}} \log P(y_{nj} = 1 | X_{n}; \boldsymbol{W}) \\ & -& (1 - \beta_{n}) \sum\limits_{j \in Y_{n}^{-}} \log P(y_{nj} = 0|X_{n}; \boldsymbol{W}), \end{array} $$

where

5 $$ P(y_{nj} = 0|X_{n}; \boldsymbol{W}) = 1 - P(y_{nj} = 1 | X_{n}; \boldsymbol{W}). $$

In these equations, *Y*_*n*_ = {*y*_*nj*_,*j* = 1,...,|*X*_*n*_|},*y*_*nj*_ ∈{0,1} denotes the matrix of the true pixel-wise labels *y*_*nj*_. The quantity ***W*** denotes the weights of the CNN which are updated during training. There are substantially more foreground pixels $Y_{n}^{-}$ in a given image than there are background pixels $Y_{n}^{+}$. Class balancing multipliers *β*_*n*_ are used to handle this imbalance on a per image basis. These are defined by

6 $$ \beta_{n} = \frac{|Y_{n}^{-}|}{|Y_{n}|} $$

with

7 $$ 1 - \beta_{n} = 1 - \frac{|Y_{n}^{-}|}{|Y_{n}|} = \frac{|Y_{n}| - |Y_{n}^{-}|}{|Y_{n}|} = \frac{|Y_{n}^{+}|}{|Y_{n}|}. $$

In the latter equations, |⋅| denotes the number of elements in the set. The CNN computes the probability *P* that a given pixel belongs to a certain class. The complementary probability is given by 1 − *P*. The pixel-wise probabilities *P* are computed by a sigmoid function to the prediction matrix obtained from the feature volume via the linear combination layer

8 $$ P(y) = \sigma (y) = \frac{1}{1 + e^{-y}}, $$

which can be implemented as a 1 × 1 convolutional layer, cf. Fig. 3. For an entire data set, the mean class-balanced cross entropy is given by

9 $$ C(\boldsymbol{W}) = \frac{1}{N} \sum\limits_{n=1}^{N} C_{n}(\boldsymbol{W}). $$

It should be noted that using two distinct CNNs for two different segments with the remaining voxels being assigned to a third class may lead to non-unique classifications of border voxels, i.e., undesirable overlaps between segments may exist. However, as will be shown in Section 3, the current approach is sufficient to accurately perform binary-class segmentation.

#### Class balancing

To identify the different regions of a nasal cavity, CNNs for multi-class segmentation are developed. In this method, a vector of class probabilities is obtained for each voxel by linearly combining the feature volume for each class. The specific class is given by the argument of the vector element with the maximum value. The CNNs are trained to identify 9 classes, i.e., the left and right frontal and sphenoid sinuses, the left and right maxillary sinuses combined, the nasal and oral cavity combined, air outside of the head and inside the mastoid air cells, bone, and tissues. Since each class is only found in a limited number of CT slices different classes are combined to reduce the total number of classes. As the distance between the left and right maxillary sinuses is large enough to assume they are never connected to each other directly, they are combined into a single class. The sides can then easily be separated once a segmentation is obtained. The left and right sides of the frontal and sphenoid sinuses are only separated by a thin bone wall, i.e., careful manual segmentation is required to generate viable training data. For some patients, the oral cavity is visible in some axial slices. Both oral and nasal cavity are combined into a single segment as the former can be removed later if needed. The air outside the head and inside the mastoid air cells is not of interest. It is specified as a distinct segment and removed.

Table 3 shows the percentage of the total number of pixels for each of the 9 segments in the unbalanced and balanced training sets. Details on the training sets are listed in Table 2.

**Table 3:** Percentage of total pixel count in the training set for 9 segments in the unbalanced and balanced case. The entries in red indicate the sinus segments, which are roughly equiprobable after balancing

	Frontal sinus left	Frontal sinus right	Maxillary sinuses	Sphenoid sinus left	Sphenoid sinus right	Nasal and oral cavities	Air outside	Bone	Tissues
Unbalanced	0.37	0.26	1.91	0.22	0.27	1.91	44.36	7.29	43.42
Balanced						1.54	45.26	6.07	44.85

The four non-sinus segments, i.e., nasal and oral cavities, air outside, bone, and tissues, make up about 97% of the total pixels, leaving approximately 3% for the 5 sinus segments. This imbalance results in CNNs that learn to classify large-share segments, but have problems learning segments with smaller shares. To increase the probability that a CNN learns all 9 classes, a balancing algorithm is applied. This algorithm minimizes the mean absolute difference between the total pixel count of each of the 5 sinus segments by repeatedly determining which sinus segment is least dominant. It finds images in the training set that contain pixels of this segment, translates or horizontally flips them (adjusting the sinus segments in the latter case, assuming bilateral symmetry), and adds them to the training set. Table 3 also shows the impact of this balancing approach on the share of the individual segments. After balancing, each of the 5 sinus segments makes up roughly 0.5% of the total number of pixels. The four remaining non-sinus segments hence make up about 97.5% of the pixel count. With the 5 sinus segments being approximately equiprobable, a given CNN is more likely to learn all 9 segments.

Additionally, two other class balancing techniques are used. First, class balancing multipliers that take into account the total number of pixels per segment in each image are used to assign higher weights to segments with a small pixel share. Second, the focal loss introduced by Lin et al. [27] is used to arrive at the balanced focal loss (BFL) function

10 $$ \begin{array}{@{}rcl@{}} BFL_{n}(\boldsymbol{W}) = -\sum\limits_{s=1}^{9} \sum\limits_{j \in {{Y_{n}^{s}}}} &&\beta_{{{Y_{n}^{s}}}} (1 - P(y_{nj}^{s} = 1 | X_{n}; \boldsymbol{W}))^{\gamma} \\ &&\log P(y_{nj}^{s} = 1 | X_{n}; \boldsymbol{W}), \end{array} $$

where $P = P(y_{nj}^{s} = 1 | X_{n}; \boldsymbol {W})$, *s* is the class index, and *γ* > 0 is a modulating factor. The BFL is more briefly expressed as

11 $$ BFL_{n}(\boldsymbol{W}) = -\sum\limits_{s=1}^{9} \sum\limits_{j \in {{Y_{n}^{s}}}} \beta_{{{Y_{n}^{s}}}} (1 - P)^{\gamma} \log P. $$

The class balancing multipliers $\beta _{{{{Y_{n}^{s}}}}}$ are defined by

12 $$ \beta_{{{Y_{n}^{s}}}} = \exp \left( -{\frac{|{X_{n}^{s}}|}{|X_{n}|}}\right), $$

where $|{X_{n}^{s}}|$ denotes the number of pixels belonging to segment *s*. That is, the pixels of segments which are less dominant in a given image are given more weight when computing the loss. The matrix ${{Y_{n}^{s}} = \{y_{nj}^{s}, j = 1,\ldots ,|X_{n}|\}}$ denotes the matrix of the true pixel-wise labels $y_{nj}^{s} \in \{0, 1\}$ for segment *s* for each *X*_*n*_. Hence, for each segment of a manual segmentation, a matrix of ones and zeros is created. Concatenated, these 9 matrices define a tensor of order 3. This tensor gives a one-hot vector with 9 elements for each pixel, i.e., each pixel is assigned to only 1 of 9 mutually exclusive segments. ${P(y_{nj}^{s} = 1 | X_{n}; \boldsymbol {W}) \in (0,1)}$ is then the probability of pixel *j* in image *X*_*n*_ belonging to segment *s*. This probability is computed by a CNN with weights ***W***. The modulating factor (1 − *P*)^*γ*^ is used to reduce the relative loss contributed by pixels which are already classified correctly. This way, more focus is given to pixels that are misclassified or classified correctly but with a lower probability, i.e., a probability closer to *P* = 0.5 rather than *P* = 1, cf. [27]. The mean BFL over an entire data set is given by

13 $$ BFL(\boldsymbol{W}) = \frac{1}{N} \sum\limits_{n=1}^{N} BFL_{n}(\boldsymbol{W}). $$

#### Multi-class segmentation

For a 9-class segmentation, the data set listed in Table 2 is used. The data set is split into training, validation, and test sets consisting of 511, 34, and 34 images, respectively. After balancing, the training set consists of 4,094 images. In total, 18 different combinations of the 9 segments are present in the entire data set. The validation and test sets include at least one of each of these combinations if possible.

The specific architecture of the network consists of multiple convolutional layers, each followed by a rectified linear unit (ReLu) activation, and a pooling layer. The latter performs max pooling and uses a kernel with both height and width of two, both height and width stride of two, and zero padding. Each pooling layer is followed by its own specialized convolutional layer, cf. Fig. 3 and a ReLu activation. The outputs are resized to the full image size using resize layers that perform bilinear interpolation without corner alignment. A concatenation of the resize layers’ outputs along the depth dimension results in a feature volume. Linear combination of the feature volume along the depth dimension then results in a prediction matrix of the same size as the original image. For each of the 9 classes, a different linear combination is computed from the feature volume. The 9 resulting prediction matrices are then concatenated along the depth dimension to obtain a prediction tensor of order three. A softmax function is applied along the depth dimension of the tensor to obtain 9 values *p*_*i*_ ∈ (0,1), which can be interpreted as class probabilities and hence obey ${\sum }_{i=0}^{8} p_{i} = 1$. Applying $\arg \max \limits (p_{i})$ gives the class predicted for a given pixel. Such segmentations can, e.g., be stored in the NRRD file format (http://teem.sourceforge.net/nrrd/format.html) used by 3D Slicer. Subsequently, the segmentations can be used to generate polygonal meshes in STL or PLY format using the Marching Cubes algorithm [25, 38]. For a detailed description on how to arrive at geometries suited for numerical simulations the reader is referred to [14, 36].

### Numerical simulation

The simulation of the flow in the nasal cavity follows a formalized procedure. First, a computational mesh is generated using the STL geometry obtained from the geometry acquisition step. Subsequently, the discretized equations of fluid motion are solved on this mesh using problem-specific boundary conditions. In this study, the TLB method is used for the simulation. In the following, these steps are described in more detail.

#### Mesh generation

**Fig. 4:**
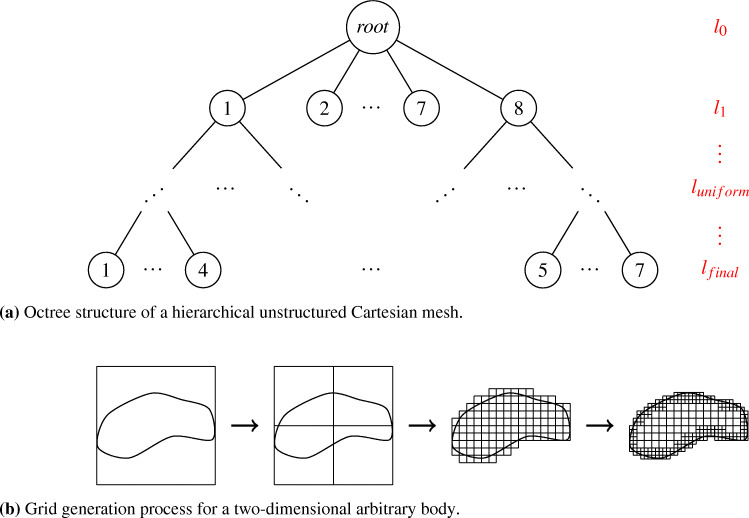
Grid generation process of m-AIA

The obtained STL files are handed to the simulation framework m-AIA. From the STL, the framework generates an hierarchical unstructured Cartesian mesh in parallel with the method described in [34]. Starting on refinement level *l*_*start*_ = 0 with an initial cube surrounding the complete geometry, the algorithm creates the computational mesh by continuously subdividing all cells of the current refinement level into 8 equally sized child cells. This subdivision processes constitutes an octree as shown in Fig. 4a. Child cells located outside the geometry are deleted. This procedure is repeated until a specified uniform refinement level *l* = *l*_*uniform*_ is reached. Afterwards, refinement patches and/or boundary refinement are created similarly until the final refinement *l* = *l*_*final*_ is achieved. The grid generation process for a two-dimensional arbitrary body including boundary refinement is shown in Fig. 4b. For more details, the reader is referred to [34].

#### Thermal lattice-Boltzmann method

A TLB method employed in this study has been previously used in [1, 14, 23, 28, 31, 32, 35, 36] to simulate respiratory flows. The code has been extensively validated in [15, 17, 31]. In this method, the discrete Bhatnagar-Gross-Krook (BGK) equation [3] of the Boltzmann equation

14 $$ f_{i}(\mathbf{r} + \boldsymbol{\xi}_{\boldsymbol{i}} \cdot \delta t, t + \delta t) = f_{i}(\mathbf{r}, t) + \omega \cdot \left( F_{i} - f_{i}(\mathbf{r}, t)\right)  $$

is solved, where *f*_*i*_ is the discrete particle probability distribution function (PPDF) in the discrete direction *i*, ***ξ***_***i***_ is the discrete molecule velocity, *t* and *δt* are the time and the time increment, and **r** is the spatial location. The spatial discretization is based on the D3Q27 model [41], i.e., 27 directions are used in three dimensions. The complex collision term of the Boltzmann equation is simplified in the BGK-model by a linear collision term with the collision frequency *ω*. The Maxwell equilibrium distribution function

15 $$ F_{i} = \rho t_{p} \cdot \left( 1 + \frac{\xi_{\alpha} v_{\alpha}}{{c_{s}^{2}}} + \frac{v_{\alpha} v_{\beta}}{2{c_{s}^{2}}} \left( \frac{\xi_{\alpha} \xi_{\beta}}{{c_{s}^{2}}} - \delta_{\alpha\beta} \right) \right),  $$

to which the PPDFs are relaxed, depends on the density *ρ*, the isothermal speed of sound *c*_*s*_, the macroscopic velocity **v** with *α*,*β* ∈ *x*,*y*,*z*, and the Kronecker delta *δ*_*αβ*_. The quantity *t*_*p*_ is a direction-dependent scaling factor.

To determine the temperature distribution inside the nasal cavity, a second set of distribution functions, the total energy distribution function (TEDF) *h*_*i*_ are solved on the same lattice [19] by

16 $$ \begin{array}{ll} h_{i}(\mathbf{r} + \boldsymbol{\xi}_{\boldsymbol{i}} \cdot \delta t, t + \delta t) & = h_{i}(\mathbf{r}, t) + \omega_{t} \cdot \left( H_{i} - h_{i}(\mathbf{r}, t)\right)\!\! \\ &+ \left( \omega_{t} - \omega_{f}\right) \cdot \left( \xi_{\alpha} v_{\alpha} - \frac{v_{\alpha} v_{\alpha}}{2}\right) \\&\cdot \left( f_{i} - F_{i}\right). \end{array}  $$

Thus, external forcing and internal heat sources are neglected. The collision frequency *ω*_*t*_ of the transport equation of the TEDF is based on the thermal conductivity. The equilibrium distribution *H*_*i*_ for the D3Q27 model reads

17 $$ \begin{array}{@{}rcl@{}} H_{i} &=& \rho {c_{s}^{2}} t_{p} \cdot \left( \frac{\xi_{\alpha} v_{\alpha}}{{c_{s}^{2}}} + \frac{v_{\alpha} v_{\beta}}{{c_{s}^{2}}} \left( \frac{\xi_{\alpha} \xi_{\beta}}{{c_{s}^{2}}} - \delta_{\alpha\beta} \right) \right.\\&&\left.+ \frac{1}{2} \left( \frac{\xi_{\alpha} \xi_{\alpha}}{{c_{s}^{2}}} - D \right) \right) + E F_{i}. \end{array} $$

It is based on the total energy *E* = (*D*/2) ⋅ *RT* + (*v*_*α*_*v*_*α*_)/2, with *T* and *R* being the temperature and the specific gas constant of the fluid, and *D* = 3 for three-dimensional flow. The quantity *H*_*i*_ depends on the number of components of the molecule velocity vector ***ξ***_***i***_. The macroscopic conservative variables are calculated from the moments of *f*_*i*_ and *h*_*i*_:

18a $$ \begin{array}{@{}rcl@{}} \rho &=& \sum\limits_{i} f_{i} \end{array} $$

18b $$ \begin{array}{@{}rcl@{}} \rho \mathbf{v} &=& \sum\limits_{i} \boldsymbol{\xi}_{\boldsymbol{i}} f_{i} \end{array} $$

18c $$ \begin{array}{@{}rcl@{}} \rho \left( \frac{D R}{2} T + \frac{v_{\alpha} v_{\alpha}}{2}\right) &=& \sum\limits_{i} h_{i}. \end{array} $$

The pressure can be expressed by the density *ρ* using the equation of state of an ideal gas.

19 $$ p = \rho R T = \rho {c_{s}^{2}}. $$

#### Boundary conditions

For the realistic simulation of respiratory flows, different sets of boundary conditions are available in m-AIA. To realize no-slip and isothermal wall conditions for a body temperature of *T*_*B*_ = 309.15 *K* at the inner walls of the nasal cavity, the interpolated bounce-back scheme of Bouzidi et al. [4] is used for the PPDF and the scheme of Li et al. [26] for the TEDF. Depending on the application, a volume flux or a fixed pressure can be applied at the pharynx. In the former case, the target Reynolds number at the pharynx $Re_{P}=(\dot {V}_{P}/A_{P})\cdot d_{P}/\nu $ is determined as a function of the hydraulic diameter *d*_*P*_, the surface area of the pharynx *A*_*P*_, the prescribed volume flux $\dot {V}_{P}$, and the kinematic viscosity of air *ν*. The time-specific Reynolds number is calculated using the velocity extrapolated from the inner cells. Depending on the difference between target and current Reynolds number, the pressure is adapted at each time step. In the latter case, the pressure at the pharynx is calculated from the ambient pressure and the given pressure difference [32]. The velocity and the temperature are extrapolated from the inner cells in both cases. At the nostrils, a modified version of the equation of Saint-Venant and Wantzel [32, 43], i.e.,

20 $$ \rho = \left( 1 - \frac{\gamma - 1}{2 \gamma} \frac{3}{\rho_{t-1}^{2}} \left( \rho_{t-1} v_{t-1}\right)^{2}\right)^{\frac{\gamma}{\gamma - 1}}  $$

is used to calculate the updated density. The momentum *ρ*_*t*− 1_*v*_*t*− 1_ is extrapolated from the inner cells at the previous time step and the isotropic exponent is set to *γ* = 1.4 [20, 32]. The temperature at the nostrils is set to the ambient air temperature *T*_*N*_ = 293.15 *K* [32]. All setup data are written to m-AIA’ configuration files and a job is submitted with the standard submission commands of JSC’s scheduler slurm.

### In-situ post-processing

The final step of the simulation pipeline is the post-processing of the raw simulation data and the visualization of the results. At run time, the simulation data are processed by m-AIA. Thus, distilled information tailored for surgery planning can be presented in-situ at run time and in a summarized manner subsequent to the simulation. In the following, first the methodologies used to process the data to be displayed in the web-based Jupyter front-end are presented before details on the various methods to analyze the flow are given.

#### ParaView-based visualization in Jupyter

To visualize the simulation data in-situ, the tool ParaView is embedded into the Jupyter environment. The interface between m-AIA and ParaView is implemented in Catalyst, the ParaView in-situ interface. This allows an in-situ data transfer from m-AIA to ParaView.

For in-situ visualization, the data are transformed from the m-AIA internal octree-based format to the ParaView-compatible Visualization Toolkit (VTK - https://www.vtk.org) format. Snapshots of the simulation can be written to disk using Python scripts. These scripts can flexibly exploit all available ParaView functions or any self-written Python code to realize any needed visualization pipeline. The output may contain predefined rendered pictures and videos, or data files that need further processing. The in-situ transfer also enables the visualization of the data directly in Jupyter, i.e., it can be used to generate predefined images, the control of the simulation, and the interaction with the visualization directly on a website.

#### Post-processing of the data

To analyze the flow, various important fluid mechanical quantities are calculated and extracted from the simulation data. The integral difference between pharynx *P* and the left and right nostril *N*(Γ),Γ ∈{*left*,*right*} of the dynamic, static, and total pressure ${\Delta } p_{dyn}^{P:N({\Gamma })}$, ${\Delta } p_{stat}^{P:N({\Gamma })}$, and ${\Delta } p_{tot}^{P:N({\Gamma })}$, and the temperature Δ*T* and the mass flux $\dot {m}$ are calculated by

21a $$ \begin{array}{@{}rcl@{}} {\Delta} p_{dyn}^{P:N({\Gamma})} &=& \frac{1}{H_{P}} \sum\limits_{i}^{H_{P}} \frac{1}{2} (\rho v^{2})_{P,i} \\ &&- \frac{1}{H_{N({\Gamma})}} \sum\limits_{j}^{H_{N({\Gamma})}} \frac{1}{2} (\rho v^{2})_{N({\Gamma})_{j}} \end{array} $$

21b $$ \begin{array}{@{}rcl@{}} {\Delta} p_{stat}^{P:N({\Gamma})} &=& \frac{1}{H_{P}} \sum\limits_{i}^{H_{P}} \rho_{P,i}~{c_{s}^{2}}\\ &&- \frac{1}{H_{N({\Gamma})}} \sum\limits_{j}^{H_{N({\Gamma})}} \rho_{N({\Gamma}),j}~{c_{s}^{2}} \end{array} $$

21c $$ \begin{array}{@{}rcl@{}} {\Delta} p_{tot}^{P:N({\Gamma})} &=& {\Delta} p_{dyn}^{P:N({\Gamma})} + {\Delta} p_{stat}^{P:N({\Gamma})} \end{array} $$

21d $$ \begin{array}{@{}rcl@{}} {\Delta} T^{P:N} &=& \frac{1}{H_{P}} \sum\limits_{i}^{H_{P}} T_{P,i} - T_{B} \end{array} $$

21e $$ \begin{array}{@{}rcl@{}} \dot{m}(L) &=& {\sum}_{i}^{H_{L}} (\rho_{0} v)_{L,i}. \end{array} $$

Here, *H* is the total number of boundary cells for the considered boundary and *L* ∈{*P*,*N*(*left*),*N*(*right*)}. To minimize numerical fluctuations in the results, a moving temporal averaging is used

22 $$ \overline{a}_{t} = \frac{1}{\Delta t + 1} \sum\limits_{k=-\frac{\Delta t}{2}}^{\frac{\Delta t}{2}} a_{t+k}, $$

where *a* is the quantity to be averaged, Δ*t* is the averaging interval, and *t* is the current time step number.

**Fig. 5:**
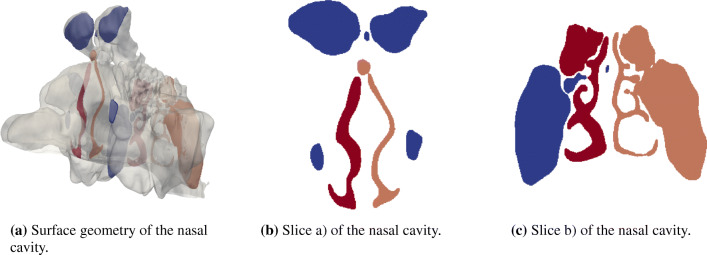
Nasal cavity containing two slices for the pressure averaging. The left and right cavities are marked in red and orange. The sinuses, which are not directly connected to the main cavity in the cross section, are colored in dark blue

Subsequent to the simulation, several post-processing tools are automatically applied to reduce the amount of raw data and to simplify the analysis for the physician. Cross sections at predefined, characteristic locations are created to provide a comprehensive overview of the flow field variables. Prior to a simulation, geometric centerlines are created for the whole nasal cavity and for each side Γ using the Vascular Modeling Toolkit (VMTK - http://www.vmtk.org). In m-AIA, cross sections orthogonal to those centerlines are created with equidistant spacing. In each cross section, the integral pressure is calculated. To distinguish between the individual Γ and parts of the sinuses, which are in the cross sections not directly connected with the main cavity, a region growing algorithm is used. Starting from the cell that contains the start point on the centerline **C**_*cl*_, neighbor cells are recursively marked, if their cell center **x** is located inside a cuboid with the thickness $\sqrt {3} \delta x$, i.e.,

23 $$ \left[\left( \mathbf{x} - \mathbf{C}_{cl} \right) \pm \frac{\sqrt{3}}{2} \delta x\right] \cdot \mathbf{n}_{cl} < 0  $$

holds using the normal vector **n**_*cl*_. Thus, only cells directly connected to the start point on the centerline are found, which allows for separating the left and right side of the main cavity and also the sinuses as shown exemplarily in Fig. 5. For those cells, the average total pressure is calculated using the spatial averaging scheme

24 $$ \overline{a}_{x} = \frac{1}{H_{cs}} \sum\limits_{i}^{H_{cs}} a_{i} $$

with *H*_*cs*_ being the total number of cells belonging to a cross section.

## Results

**Fig. 6:**
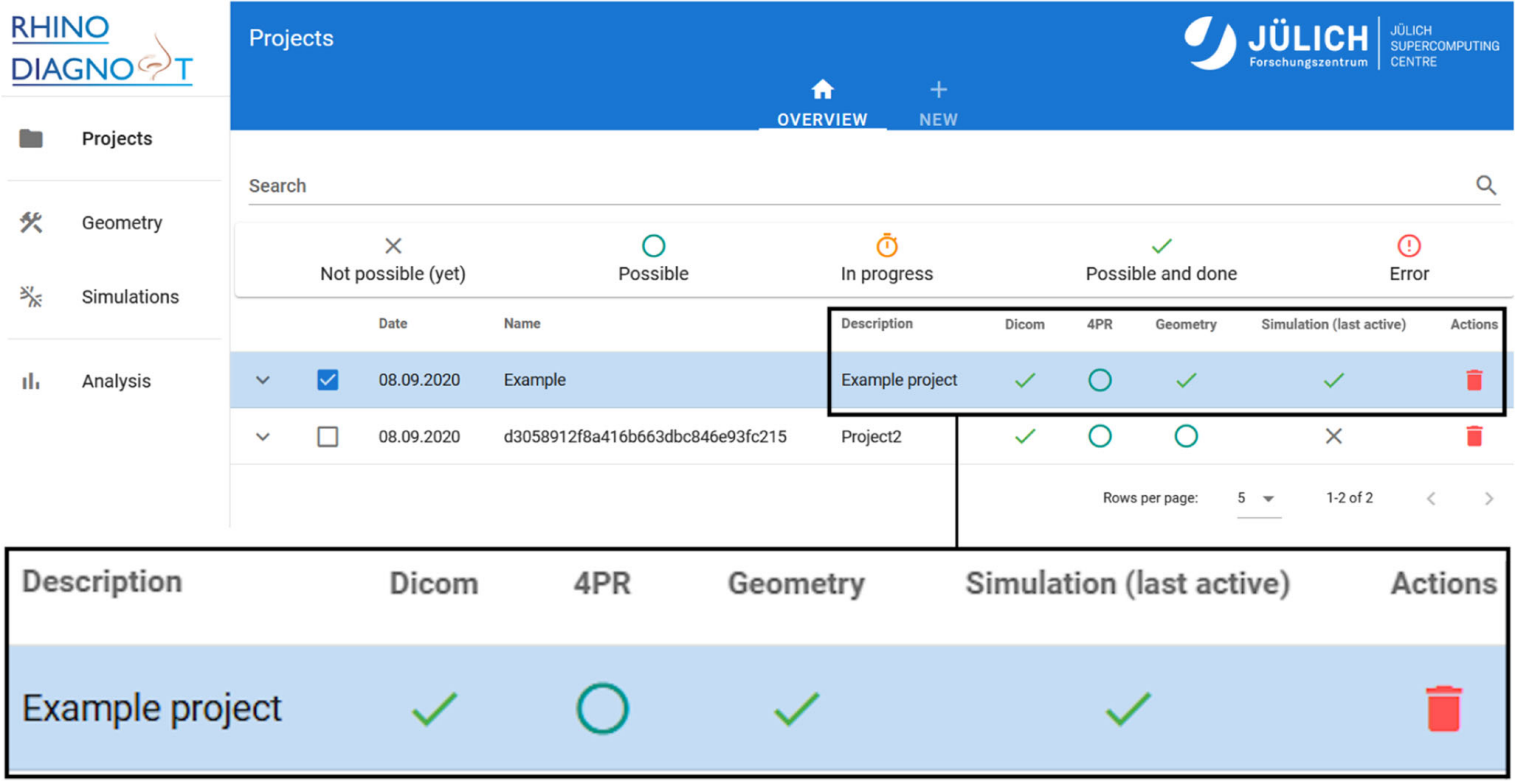
The starting menu of the web application allows the creation of a new or to edit an existing project. The inset shows a zoom of the project view. The status of the pipeline steps is indicated by different icons

In the following, the functionality of the Jupyter-based framework for the simulation of respiratory flows and interactive supercomputing, cf. Fig. 1, is demonstrated on the basis of an exemplary pathological case. That is, the results of the individual pipeline steps, from the ML-based geometry acquisition over 4-PR measurements to the final post-processed simulation data, are presented. The starting menu of the web application is shown in Fig. 6. After creating a new project, the user is asked to upload CT and if available 4-PR measurement data. Subsequently, the simulation pipeline is started. The current status of the various pipeline steps is indicated by icons in the starting menu. In the subsequent presentations, the left and right side correspond to those from a patient’s point of view.

### Visualization of 4-PR measurement results

**Fig. 7:**
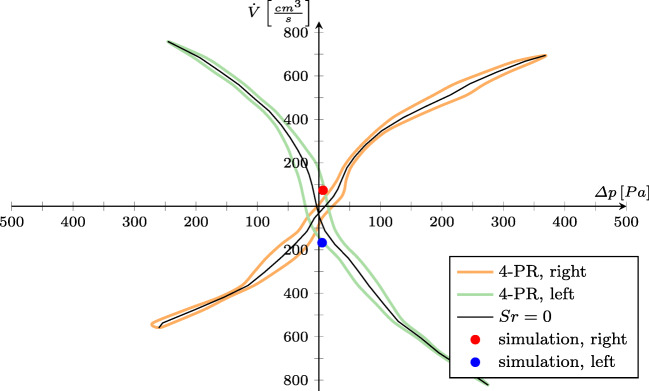
Results of the 4-PR measurements for inspiration and expiration. Furthermore, the steady state curve for *Sr* = 0, overlayed by the simulation results, is shown

Prior to starting the automated flow analysis, the user has the opportunity to upload 4-PR measurement data of the investigated patient along with the corresponding CT data. This allows to collect all patient-specific data in a single project. The visualization of these data complements the results of an analysis of steady flow cases. In Fig. 7, the plot of the 4-PR measurement data is presented such that is common to rhinologists, i.e., the volume flux in $\left [cm^{3}/s\right ]$ is plotted over the pressure loss in $\left [Pa\right ]$ for inspiration and expiration, cf. Fig. 2b. In addition to these two curves, the case for Strouhal*Sr* = *ω*_*resp*_ ⋅ *d*_*p*_/*v* = 0 representing steady simulations with a respiration frequency of *ω*_*resp*_ = 0 is shown. When the results from a numerical simulation become available, the graph is extended by the steady state results (red and blue dots).

Note that the non-perfect alignment of the simulation results with the pressure curve from the measurements for $Sr \rightarrow 0$ is caused by different swelling states of the nasal cavity at the measurement and CT-recording times.

Furthermore, it should be noted that the environment is currently extended to use the 4-PR data in the boundary conditions to excite unsteady flow, see Section 5.

### Segmentation with CNNs

Segmentations are performed using both the binary- and multi-class methods. Next, the corresponding results are presented.

#### Results of binary-class segmentations

The best models for binary air segmentation that employ the hyperparameters specified in Section 2.3 reach a validation accuracy of up to 99.5%. This is the only metric used for the binary models trained. A grid search is performed with learning rates in the range of [10^− 7^,10^− 1^]. The batch size is set to 1. The Adam optimizers are used for cost optimization [24]. Network weights are initialized using random values from a truncated normal distribution. In the computation of the cost, all pixels are either weighted equal or pixels are assigned a higher weight if they are located in an image region where intensity changes quickly such as in transition regions, e.g., between the air-filled inner region of a cavity and the bone surrounding it. The grid search produces similar models in terms of the final validation accuracy and of the segmentation quality as determined by subjective visual inspection. That is, the accuracy does not improve as compared to the best models mentioned above. The same process is used to obtain models for bone segmentation, which results in a validation accuracy of up to 96.82%. Note that these accuracy values by themselves do only tell how similar the CNN segmentation results are to the manually segmented data, which serve as ground truth. They only provide additional information on the segmentation quality in general if the manual segmentations are highly accurate. Testing the trained models on unseen patient CT data, the segmentation using both the air and bone classifiers yield similar results to those obtained after training and validation as judged by careful visual inspection. This even holds for those models trained and validated with only the 213 axial slices of a single patient, cf. Table 2.

**Fig. 8:**
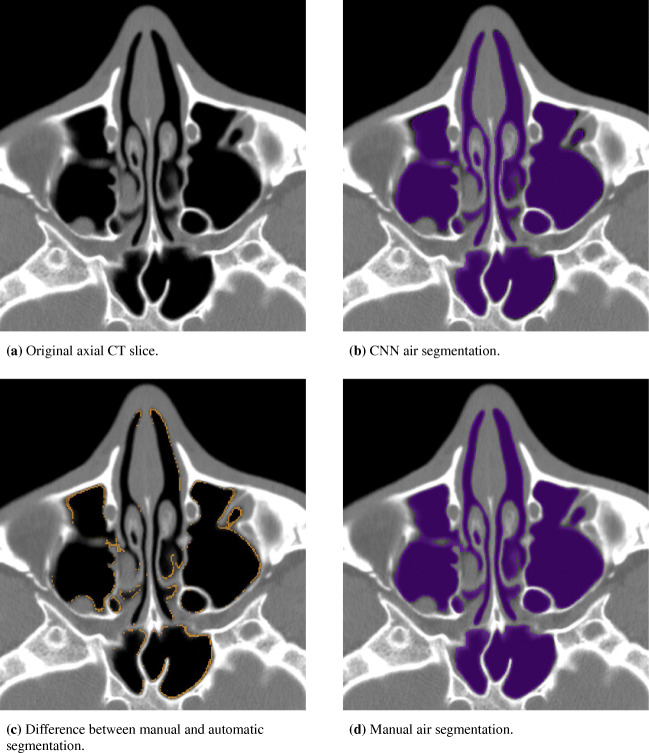
Comparison of the results from CNN and manual air segmentation

In Fig. 8, the results of automatically and manually segmented data are shown for a single axial CT slice. The original slice is depicted in Fig. 8a, while Figs. 8b and d show the automatically and manually segmented data. Evidently, the results are almost identical. However, the CNN appears to be a bit more conservative than the trained person at the boundaries of the cavities and at air to tissue transitions between adjacent axial slices. Figure 8c highlights the segmentation difference.

#### Results of multi-class segmentations

The segmentation of different air-filled parts of a CT image cannot be performed on the basis of Hounsfield unit ranges alone. A CNN is capable of performing this task by taking into account the 2- or 3-dimensional environment of a given voxel. It performs a classification based on possibly thousands of environments it was trained on. This enables to, e.g., remove the air outside of the head and extract the individual paranasal sinuses automatically.

The most accurate 9-class segmentation CNN in this study achieves test and validation accuracies of 97.17% and 96.91%. Further metrics are used to assess the quality of the segmentations. The first metric is the average Jaccard index $\mathcal {J}$ (intersection over union). This metric is used to measure the overlap of corresponding segments between manual and CNN segmentation with $\mathcal {J}=100\%$ representing a perfect match. It is determined by computing the Jaccard index for each of the 9 segments for each image, and averaging over all 9 segments and over the entire test set. For this set, an average Jaccard index of $\mathcal {J}=86.29\%$ and an average segment match of $\mathcal {S}=97.39\%$ is obtained. The latter metric is determined by computing how many segments out of 9 classes are present in both the manual and CNN segmentation for each image and then averaging over all images in the test set. The segment match $\mathcal {S}$ quantifies if the segments found in the manual and CNN segmentations are the same, i.e., for $\mathcal {S}=100\%$, the segments predicted by the CNN for all images in a given data set are exactly those, which are present in the images according to the manual segmentations. Smaller values of $\mathcal {S}$ indicate that the CNN predictions are wrong or that too few or too many classes have been predicted. Another metric used is the averaged connected component match $\mathcal {C}$, which is determined by computing the absolute difference in connected component count between manual and CNN segmentation for each segment of an image, averaging over all segments of the image, and averaging over all images of the test set. A value of $\mathcal {C}=100\%$ indicates that the numbers of connected components in the manual and CNN segmentations are exactly the same in every image of the test set. For the given test set, a value of $\mathcal {C}=69.11\%$ is found.

**Fig. 9:**
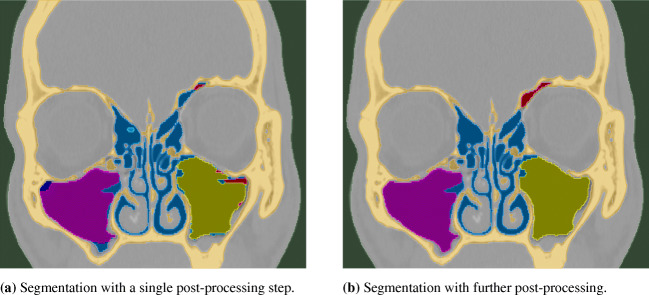
Multi-class CNN segmentation with and without post-processing for a single CT slice

Figure 9 shows the segmentation generated by a 9-class CNN for an arbitrary 400 *px*^2^ ROI on an axial CT slice. In Fig. 9a, the result of automatically applying a single post-processing step that splits the combined maxillary sinuses segment into left and right components, yielding 10 different classes, is shown. In contrast, Fig. 9b shows the segmentation after two more post-processing steps. In the first additional step, each voxel of connected components with less than 10 voxels is given the dominant color of its 3 *px*^3^ neighborhood. The second step assigns correct colors to anatomically implausible components. For example, the small dark blue component shown in Fig. 9a in the right frontal sinus is recolored pink in Fig. 9b. Note that the last post-processing step is only applicable if the patient’s geometry is deemed healthy, which here means that each of the cavities are given by single connected components.

**Fig. 10:**
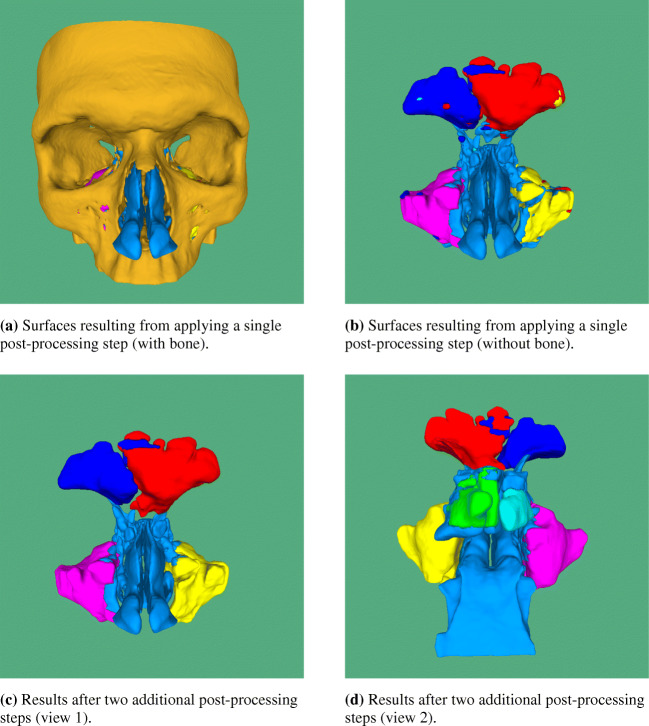
STL geometries generated from a multi-class CNN segmentation using a single and two further post-processing steps

On a standard desktop computer (CPU: IntelCore i7-7820X with 16 cores clocked at 3.60GHz, GPU: GeForceGTX 1080/PcIe/SSE2, Memory: 64GB), a segmentation in NRRD format and 3D geometries in STL and/or PLY format can be generated in approximately 3 min for single patient data consisting of ≈ 200 axial slices using a 400 *px*^2^ ROI. The complete process roughly takes 8 min when the two additional post-processing steps are included. Figure 10 shows 3D geometries generated from the 2D segmentations with the single and the two additional post-processing steps. In Fig. 10a, the bone geometry is also shown, which is removed for all other cases for visualization purposes. By comparing Fig. 10b with the two views of the same corrected geometry in Fig. 10c and d, it becomes clear that without applying the two additional post-processing steps, the sub-segmentation is less accurate. The sphenoid sinuses appear to be smaller than they should be in the ideal case. A single connection exists between the frontal sinuses due to limited segmentation accuracy at the thin bone wall separating them. The results obtained for the maxillary sinuses appear to be particularly accurate. Nevertheless, the CNN evidently already learned to discern the sinuses quite well despite the fact that it is only trained on data of three healthy patients.

### Simulation of the respiratory flow

Subsequent to the geometry acquisition, the framework guides the user towards the simulation setup. After completing the setup, the simulations can be executed and the results can be displayed and analyzed. These steps are explained in the following.

#### Simulation preparation

**Fig. 11:**
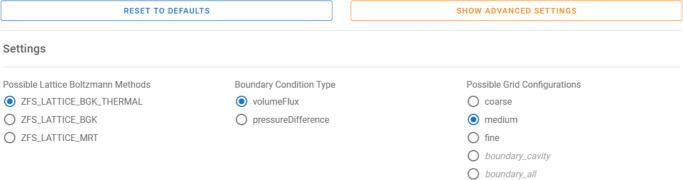
In the simulation setup the user can choose from several configurations. Here, the simulation method, the types of boundary conditions, and the grid refinement can be selected. Advanced users can set additional configuration options

In the setup process, the simulation parameters can be specified in simplified drop-down menus in the Jupyter framework as shown in Fig. 11. The user can choose between different grid refinement levels, different simulation parameters, and boundary conditions. Default values are pre-selected.

For the simulation of the sample nasal cavity, a uniformly refined, medium fine mesh with a grid cell size of *δr* = 0.106 *mm*, which results in approximately 100 ⋅ 10^6^ cells, is generated. Considering quasi-steady flow at moderate volume fluxes leads to Reynolds numbers *Re*_*p*_ < 2000.

In this regime, the flow is neither fully laminar nor turbulent [21]. It has been shown in [32] that for such conditions the employed resolution is sufficient to resolve all turbulent structures impacting the fluid mechanical properties of respiration.

**Fig. 12:**
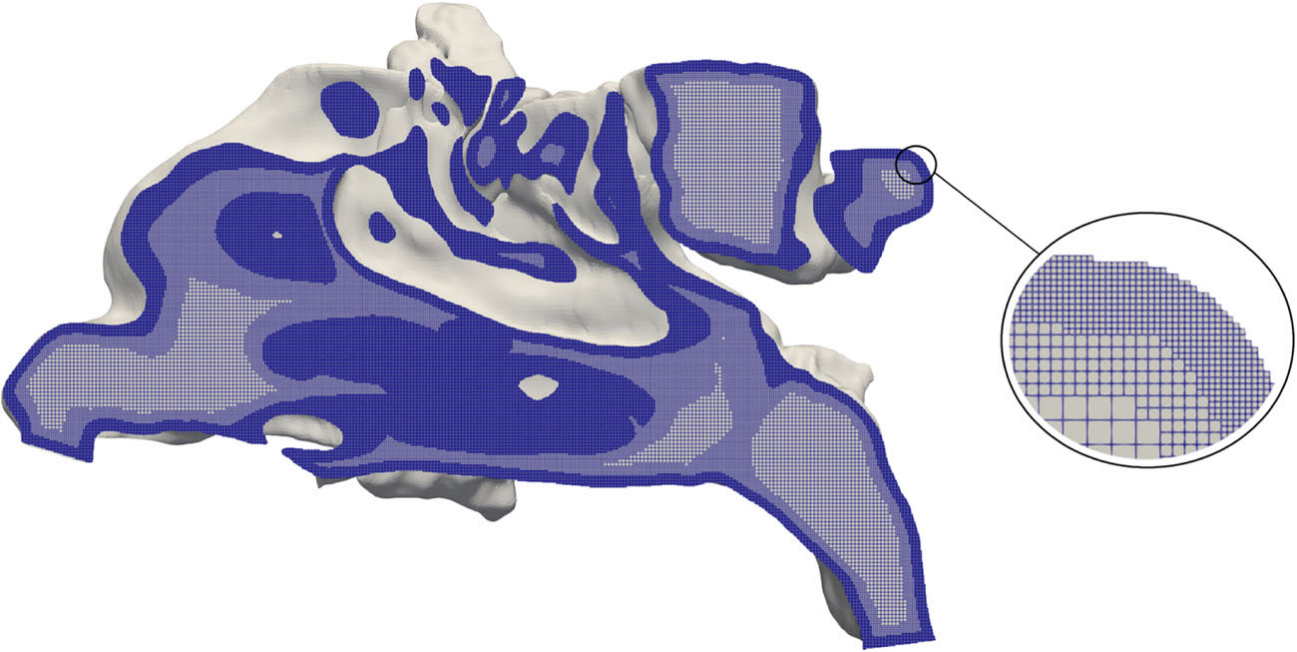
Hierarchical unstructured Cartesian mesh for the simulation of the flow in the nasal cavity. A boundary-refined mesh is shown to emphasize the capabilities of the parallel meshing tool in m-AIA

A computational mesh, as output of the massively parallel grid generator of m-AIA is shown in Fig. 12. For demonstration purposes, the example mesh also features boundary-refined elements. The flow in the frontal sinuses does not have a significant effect on the respiratory flow through the main cavities. Therefore, and to save computational resources, the frontal sinuses are removed.

The boundary conditions are set as described in Section 2.4.3, i.e., a volume flux of $\dot {V} = 250~ml/s$ is prescribed for the steady state simulation. This value corresponds to the averaged volume flux for respiration at rest for an adult. Note that this is only the default value. The user can prescribe any physically valid volume flux. However, depending on the volume flux a higher grid resolution might be required.

The corresponding Reynolds number is calculated automatically using the hydraulic diameter of the pharynx *d*_*h*_ and the kinematic viscosity of air *ν* = 1.63 ⋅ 10^− 5^*m*/*s*^2^ at the average ambient air temperature of *T*_*avg*_ = 303.15 *K*.

#### Simulation run and in-situ monitoring

**Fig. 13:**
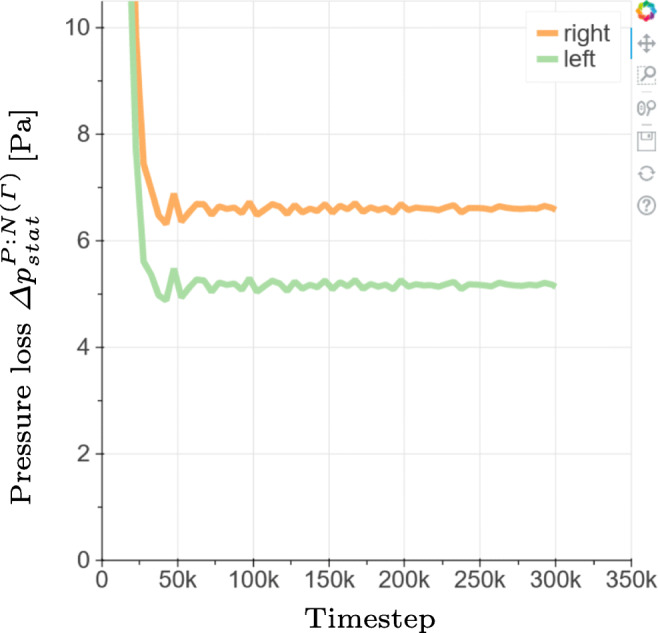
Temporal evolution of the static pressure loss plotted in-situ at simulation run time

At simulation run time, some of the flow variables such as ${\Delta } p_{tot}^{P:N({\Gamma })}$ are visualized in-situ. This allows the user to monitor the simulation. For quasi-steady simulations, as used in this study, the in-situ output of the flow variables can be used to monitor the convergence of the simulation. In contrast, if unsteady simulations are desired the temporarily resolved evolution of the flow variables will become available to the user. An example output of ${\Delta } p_{stat}^{P:N({\Gamma })}$ over the number of iterations is shown in Fig. 13. The number of iterations used for one simulation run is set to 300,000. Subsequently, the simulation is restarted to average the results over another 300,000 iterations. The user can modify both the number of the simulation and the averaging iterations in the Jupyter interface.

#### Output and analysis of the simulation results

**Fig. 14:**
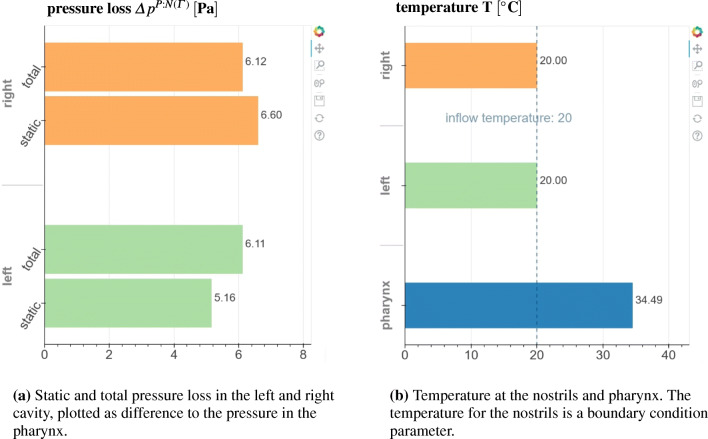
Bar diagrams of the aggregated static and total pressure loss (a) and the aggregated temperature (b), available in the web application

The averaging process is followed by the post-processing of the simulation data. In this pipeline step, the user is able to choose between different analysis tools. Initially, the Jupyter interface provides the user with an analysis of the flow variables that is presented in multiple plots. That is, the averaged temperature at the inlets and at the outlet, and the total and static pressure losses are displayed. The results for the example case are shown in Fig. 14. Furthermore, the volume flux in combination with the pressure loss is presented in the 4-PR measurement data plot in Fig. 7. These values provide the user with a first impression of the quality of the nasal cavity. From Fig. 14a, it is obvious that the pressure loss in the exemplary case is moderate. Furthermore, the heating capability determined by an averaged temperature increase up to 99% body temperature is excellent, cf. Fig. 14b.

**Fig. 15:**
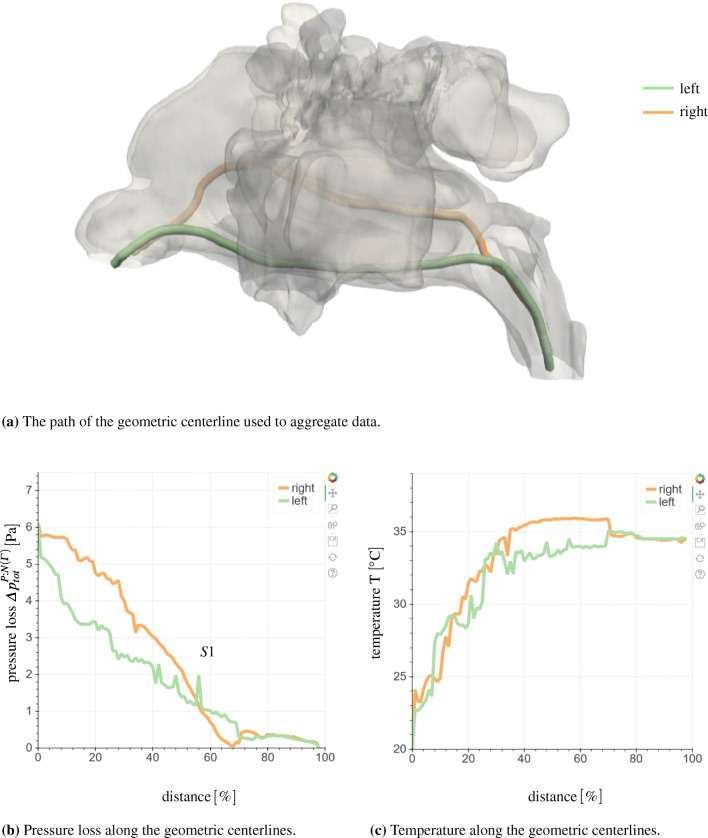
The geometric centerline inside the nasal cavity can be visualized in the web application, cf. (a). The flow variables like the pressure or the temperature are plotted along the centerlines, starting from the left and right nostrils. The distance from the nostrils is shown in percentage of the total length of the centerline

**Fig. 16:**
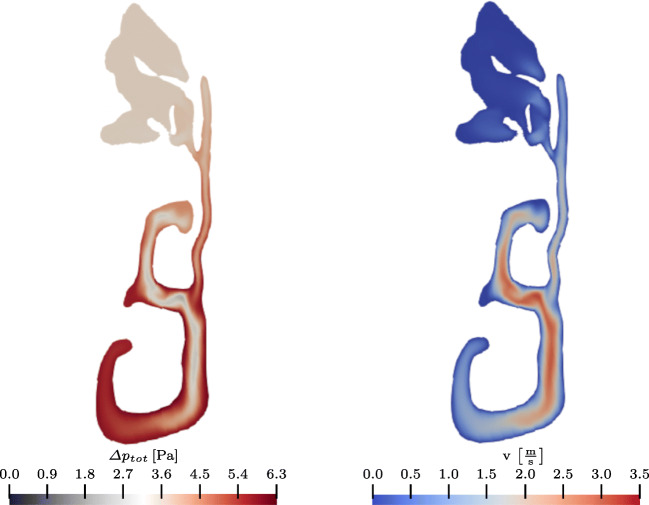
Total pressure difference between the pressure at the inlet and the cross section *S*1, cf. Fig. 15b. Furthermore, the velocity magnitude distribution at location *S*1 is shown

**Fig. 17:**
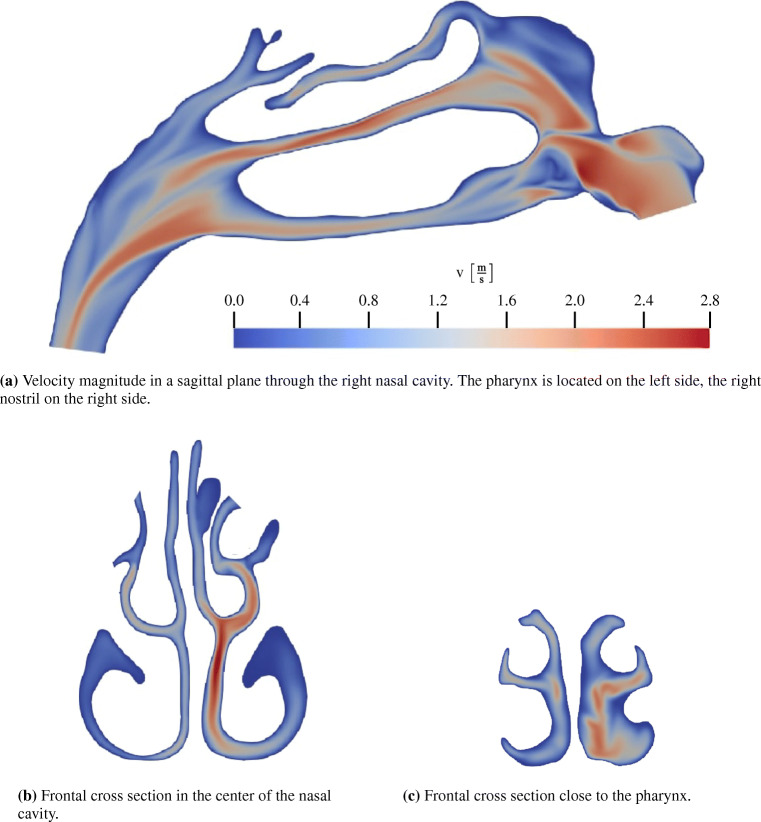
Sagittal and frontal cross sections colored by velocity magnitude

For a more detailed view, the pressure and temperature evolution along the geometric centerline is presented to the user. The geometric centerline of the investigated nasal cavity is shown in Fig. 15. Its computation is based on the explanations given in Section 2.5.2. The pressure and temperature along this curve are shown in Fig. 15b and c, respectively. Using this data representation, the user can easily identify regions of high pressure loss or further conspicuous areas without searching the whole simulation data. The peak *S*1 shown in Fig. 15b is such a candidate. After localizing these critical regions, the user can interact with the Jupyter interface to visualize the flow variables in these regions. It is furthermore possible to visualize other regions or even the whole computational domain. Therefore, the simulation data is rendered in parallel on the visualization nodes of the JURECA supercomputer using ParaView. Figure 16 shows the cross section corresponding to *S*1. Obviously, the cross section cuts a narrow passage between the frontal sinus and the main cavity. The total pressure within the frontal sinus is greater as the total pressure in the main cavity, which results in the peak *S*1 in Fig. 15b. Furthermore, this pressure difference between the frontal sinus and the main cavity can cause pain that might be alleviated by decongestants that widen the passage. Figure 17 shows further rendered data in the web application. Here, sagittal and frontal cross sections with the velocity magnitude are shown. Obviously, and in accordance with the reduced pressure loss in the left nasal cavity, accelerated flow is found on the left side.

## Discussion, summary, and conclusions

CFD methods slowly make their way into clinical environments. One of the biggest challenges that needs to be addressed is finding a suitable pipeline that (i) delivers reliable results, (ii) can be executed efficiently and fully automatically, (iii) is intuitively usable by medical experts, and (iv) is easily extendable by software developers.

The approach presented in this study fulfills all theses requirements and focuses on the implementation and deployment of a fully automated simulation pipeline for decision making processes and for surgery planning in the field of rhinology. The automatization is facilitated by including ML methods into the pipeline. The desired accuracy is guaranteed by running scale-resolving simulations without using any modeling aspects. Results are obtained in a short amount of time through the use of HPC systems. The usability and the extensibility of the software framework is provided through using a Jupyter-based framework. It allows for intuitive data progressing, a clear presentation of the results, and easy access to HPC resources hiding the whole complexity of running numerical simulations from the user. The pipeline is executed on JSC’s HPC systems, can be transferred, however, to any other system supporting JupyterLab with a voilà extension and having access to sufficient computing resources.

Initially, the pipeline accepts CT data sets, which are anonymized on the client side, i.e., no personal data is treated on the server side. Additionally, 4-PR data can be uploaded and visualized, after simulation even with numerical results integrated. Subsequently, novel CNN-based algorithms are capable of either performing a two-class binary segmentation of the airways or a 9-class segmentation identifying several relevant anatomical structures. In the former case, which is the most relevant case when considering numerical simulations of the respiratory flow, the air-filled regions in particular are confined fairly strictly to a certain Hounsfield unit range. For the latter case, it is difficult to classify a given voxel, regardless of whether the segmentation is performed manually by a person or automatically. That is, bones or tissue can be difficult to discern in regions where the Hounsfield units of different materials are practically indistinguishable. In this work, two CNNs have been developed that are capable of providing accurate results for binary-class segmentations and to classify nine different anatomical structures. The multi-class segmentation algorithm is enhanced by class balancing methods. All CNNs have been trained by multiple CT data sets and deliver a segmentation in a short amount of time with a sufficient accuracy, even on single desktop computers. From these segmentations water-tight geometries are obtained by standardized techniques.

These surfaces are input to a grid generator, which creates a hierarchical, unstructured Cartesian mesh to run numerical simulations. From the novice user, advanced options that, e.g., influence the mesh resolution, are hidden in the configuration process. They are set to decent standard values. The advanced user has, however, the opportunity to influence the mesh generation by, e.g., modifying the maximum refinement level or the refinement type. Since the mesh generation process requires less computational resources than a simulation, the job is in general executed within a few seconds.

On the computational mesh, the governing equations of a TLB method are solved, i.e., the BGK equation and its thermal extension by a total energy distribution function approach are solved. Unlike other solvers employing RANS methods, in which turbulent structures are only modeled, using the default option of the pipeline, the mesh is sufficiently fine to fully resolve all relevant flow phenomena without using any model. The TLB method yields highly resolved flows, which are required for an accurate medical analysis. Obviously, this comes at a high computational cost. On single desktop computers, results cannot be obtained in a reasonable amount of time. Fortunately, the Jupyter interface has been designed in such a way to directly grant access to HPC resources through allocated compute time projects. Thereby, simulations can be executed in a massively reduced amount of time using thousands of computational cores. The simulation framework m-AIA, especially its TLB component, has shown to scale to hundreds of thousands of computational cores, i.e., it is expected that similar simulation problems can simultaneously be solved in only a fraction of the time on soon upcoming exascale systems.

The complete setup of the simulation is integrated into the Jupyter framework. Like in the grid generation process, the framework provides a simplified interface pre-filled with decent default values, i.e., the user not necessarily has to deal with the complex choice of reasonable boundary conditions, simulation times, or the parallelism of the execution. Various drop-down menus offer meaningful options for the prescription of boundary conditions. For example, the user can simply choose from prescribing a volume flux or driving the flow by a pressure difference from the nostrils to the pharynx. Hence, in contrast to other research codes that are used to conduct highly resolved simulations of the respiratory system, the user does not require any knowledge on HPC and CFD to run simulations using the proposed pipeline. In any case, there also exists an advanced users’ view. Here, the user may, e.g., additionally choose from various discretization models or influence the parallel simulation execution. The submission to the batch system and the execution is completely hidden behind a single simulation run button. Internally, a job script is modified, the properties of the simulation are written to m-AIA property file and a job is submitted. Of course the execution time is dependent on the availability of the requested resources. However, for the small number of cores as considered here, the queuing takes in general only a few minutes.

At run time, some of the simulation data is already available in-situ and is visualized in the Jupyter framework. That is, the pressure loss and the temperature difference are presented to the user in form of plots. At this stage of the pipeline, they serve as monitoring functions for the convergence of the simulation in case of steady state simulations. In unsteady simulations, the plots show the temporal evolution of the variables.

Subsequent to the simulation the result data are post-processed. The user can intuitively interact with various plots presented in the Jupyter environment. Besides standard bar plots of the flow variables pressure loss, temperature difference, and mass flux, the user is offered the distributions of the pressure loss and temperature difference along the geometric centerline of the left and right nasal cavity. This way, critical areas inside the airways, which can be investigated in detail afterwards by visualizing the rendered simulation data, can be analyzed in detail.

To summarize, the Jupyter platform with the voilà extension is a perfect tool to link all the individual steps that are required to run numerical simulations of respiratory flows on HPC systems. It guides novice and professional users through the complex process of running such simulations and analyzing the results. The individual steps can be executed fully automatically. The interaction with the user is only required to specify additional simulation properties and to allow the user to stop the pipeline at any point. In principle, default values can be applied to execute the pipeline without any user interaction.

Thus, the pipeline is capable of combining easy usability and highly resolved simulations including automated pre- and post-processing on HPC systems, which does not yet exist in this form to the best of the authors knowledge. Furthermore, Jupyter’s flexibility allows the easy adaptation of the pipeline to any other simulation problem.

## Outlook

The next step is to integrate the pipeline into medical trials to get more intense feedback from medical experts on the usability of the framework, when integrated into everyday clinical routine. Several hospitals are already interested in joining such a study. Furthermore, the Jupyter notebooks are currently prepared to make them available to other supercomputer users of JSC. They will serve as templates for different communities to implement domain-specific notebooks to efficiently process simulation data and to advance interactive supercomputing.

Of course, there is also potential to improve the accuracy and performance of the individual pipeline steps. 3D CNNs that make use of more training data and consider information along the axial dimension will be implemented. Three-dimensional information, much larger yet accurately segmented data sets, and additional data set enhancement techniques such as scaling and translation of the volume data, will be exploited. This will lead to CNNs that provide more accurate results for a wide range of patient geometries, for both healthy and pathological cases. Such CNNs may also enable large-scale data analysis for, e.g., CFD simulations, volumetric measurements, obstruction detection, or septum deviation gradation, using thousands of patients.

Considering the simulation, m-AIA is currently extended to read 4-PR data as input and to simulate complete respiration cycles according to these data. This enables the prescription of time-resolved volume fluxes that correspond to the 4-PR data, i.e., to create fully-integrated and realistic digital twins of patients. Furthermore, an interface is implemented in m-AIA to interact with a virtual surgery environment. In this environment, a surgeon will be able to conduct virtual surgeries on segmented geometries at simulation run time, the fluid mechanics will automatically be updated, and feedback will be given to the surgeon. Therefore, the TLB method will be coupled to a level-set solver tracking the change of the air/tissue interface at virtual surgery. Furthermore, the TLB method is extended to allow for analyses of the heat transfer at this interface. That is, the impact of various inlet temperatures on the tissue temperature is investigated. To this end, m-AIA is also continuously adapted to new supercomputing architectures to prepare it for exascale computing, i.e., various modules of m-AIA are currently ported to general purpose graphics processing units (GPGPUs), further load-balancing techniques are investigated, and the modular supercomputing architectures at JSC are exploited for efficient in-situ analysis.

## Abbreviations

4-*PR*: 4-phase rhinomanometry
*BFL*: Balanced focal loss
*BGK*: Bhatnagar-Gross-Krook
*CFD*: Computational fluid dynamics
*CNN*: Convolutional neural networks
*CT*: Computed tomography
*DL*: Deep learning
*DNS*: Direct numerical simulation
*GPGPU*: General purpose graphics processing unit
*GUI*: Graphical user interface
*HPC*: High-performance computing
*I**S**C**O**A**N**A*: International Standardization Committee on the Objective Assessment of the Upper Airways
*JSC*: Jülich Supercomputing Centre
*LES* Large-eddy simulations *m* −*A**I**A*: multiphysics Aerodynamisches Institut Aachen *M**D*5 Message-digest algorithm 5
*ML*: Machine learning
*MRI*: Magnetic resonance imaging
*PNIF*: Peak nasal inspiration flow
*PPDF*: Particle probability distribution function
*RANS*: Reynolds-Averaged Navier-Stokes
*R**e**L**u*: Rectified linear unit
*RMS*: Root-mean-square
*ROI*: Region of interest
*STL*: Standard tessellation language
*TEDF*: Total energy distribution function
*TLB*: Thermal lattice-Boltzmann
*VAS*: Visual analogue scale
*VTK*: Visualization Toolkit
*VTKM*: Vascular Modeling Toolkit
*ZFS*: Zonal Flow Solver

## References

[1] Achilles N, Pasch N, Lintermann A, Schrȯder W, Mȯ,sges R (2013). Computational fluid dynamics: a suitable assessment tool for demonstrating the antiobstructive effect of drugs in the therapy of allergic rhinitis. Acta otorhinolaryngol Itali organ ufficiale della Soc italian otorinolaringol chirurgia cervico-facciale.

[2] Bates AJ, Doorly DJ, Cetto R, Calmet H, Gambaruto AM, Tolley NS, Houzeaux G, Schroter RC (2014) Dynamics of airflow in a short inhalation. J R Soc Interface 12(102):20140880–20140880. 10.1098/rsif.2014.0880 10.1098/rsif.2014.0880 PMC4277078 25551147

[3] Bhatnagar PL, Gross EP, Krook M (1954). A model for collision processes in gases. I. small amplitude processes in charged and neutral one-component systems. Phys Rev.

[4] Bouzidi M, Firdaouss M, Lallemand P (2001). M,omentum transfer of a Boltzmann-lattice fluid with boundaries. Phys Fluids.

[5] Burgos MA, Sanmiguel-Rojas E, del Pino C, Sevilla-García MA, Esteban-Ortega F (2017). New cfd tools to evaluate nasal airflow. Eur Arch Otorhinol.

[6] Calmet H, Gambaruto AM, Bates AJ, Vȧzquez M, Houzeaux G, Doorly DJ (2016). Large-scale CFD simulations of the transitional and turbulent regime for the large human airways during rapid inhalation. Comput Biol Med.

[7] Calmet H, Yamamoto T, Eguzkitza B, Lehmkuhl O, Olivares E, Kobayashi Y, Tomoda K, Houzeaux G, Vȧzquez M (2019). Numerical evaluation of aerosol exhalation through nose treatment. J Aerosol Sci.

[8] Chien KY (1982). Predictions of channel and Boundary-Layer flows with a Low-Reynolds-Number turbulence model. AIAA J.

[9] Clement P (1984). Committee report on standardization of rhinomanometry. Rhinology.

[10] Clement P, Gordts F (2005). Consensus report on acoustic rhinometry and rhinomanometry. Rhinology.

[11] Croy I, Hummel T, Pade A, Pade J (2010). Quality of life following nasal surgery. Laryngoscope.

[12] Damm M, Quante G, Jungehuelsing M, Stennert E (2002). Impact of functional endoscopic sinus surgery on symptoms and quality of life in chronic rhinosinusitis. Laryngoscope.

[13] Dworkin MJ, Barker EB, Nechvatal JR, Foti J, Bassham LE, Roback E, Dray JF Jr (2001) Advanced encryption standard (AES). 10.6028/NIST.FIPS.197 10.6028/jres.106.023 PMC4863838 27500035

[14] Eitel G, Freitas RK, Lintermann A, Meinke M, Schröder W (2010) Numerical Simulation of Nasal Cavity Flow Based on a Lattice-Boltzmann Method. In: Dillmann A, Heller G, Klaas M, Kreplin HP, Nitsche W, Schröder W (eds) New Results in Numerical and Experimental Fluid Mechanics VII, Notes on Numerical Fluid Mechanics and Multidisciplinary Design, vol 112. Springer, Berlin, pp 513–520. 10.1007/978-3-642-14243-7_63

[15] Eitel-Amor G, Meinke M, Schrȯder W (2013). A lattice-Boltzmann method with hierarchically refined meshes. Comput Fluids.

[16] Faramarzi M, Baradaranfar M, Abouali O, Atighechi S, Ahmadi G, Farhadi P, Keshavarzian E, Behniafard N, Baradaranfar A (2014). Numerical investigation of the flow field in realistic nasal septal perforation geometry. Allergy Rhinol (Providence R.I.).

[17] Freitas RK, Henze A, Meinke M, Schrȯder W (2011). Analysis of Lattice-Boltzmann methods for internal flows. Comput Fluids.

[18] Göbbert J.H, Kreuzer T, Grosch A, Lintermann A, Riedel M (2018) Enabling Interactive Supercomputing at JSC Lessons Learned: ISC High Performance 2018 International Workshops, Frankfurt/Main, Germany, Revised Selected Papers, pp 669–677. 10.1007/978-3-030-02465-9_48

[19] Guo Z, Zheng C, Shi B (2007). Thermal lattice boltzmann equation for low mach number flows: Decoupling model. Phys Rev E.

[20] Hörschler I, Brücker C, Schröder W, Meinke M (2006). Investigation of the impact of the geometry on the nose flow. Eur J Mech - B/Fluids.

[21] Hörschler I, Schröder W, Meinke M (2010). On the assumption of steadiness of nasal cavity flow. J Biomech.

[22] Jülich Supercomputing Centre: JURECA: Modular supercomputer at Jülich Supercomputing Centre. J Large-scale Res Facilities 4(A132). 10.17815/jlsrf-4-121-1

[23] Kim SY, Park YC, Lee KJ, Lintermann A, Han SS, Yu HS, Choi YJ (2018). Assessment of changes in the nasal airway after nonsurgical miniscrew-assisted rapid maxillary expansion in young adults. Angle Orthodont.

[24] Kingma DP, Ba J (2014) Adam: A method for stochastic optimization. International Conference on Learning Representations PMC13460018 42583277

[25] Lewiner T, Lopes H, Vieira AW, Tavares G (2003). Efficient implementation of marching cubes’ cases with topological guarantees. J Graph Tools.

[26] Li L, Mei R, Klausner JF (2013). Boundary conditions for thermal lattice Boltzmann equation method. J Comput Phys.

[27] Lin T, Goyal P, Girshick R, He K, Dollár P (2017) Focal loss for dense object detection. In: 2017 IEEE International conference on computer vision (ICCV), pp 2999–3007. 10.1109/ICCV.2017.324

[28] Lintermann A (2016) Efficient parallel geometry distribution for the simulation of complex flows Papadrakakis M, Papadopoulos V, Stefanou G, Plevris V (eds), Institute of Structural Analysis and Antiseismic Research School of Civil Engineering National Technical University of Athens (NTUA) Greece, Athens. 10.7712/100016.1885.5067

[29] Lintermann A (2020) Application of Computational Fluid Dynamics Methods to Understand Nasal Cavity Flows. In: Cingi C, Muluk NB (eds) All Around the Nose, chap. 9. 1st edn. 10.1007/978-3-030-21217-9_9. Springer International Publishing, Cham, pp 75–84

[30] Lintermann A, Eitel-Amor G, Meinke M, Schröder W (2013) Lattice-Boltzmann Solutions with Local Grid Refinement for Nasal Cavity Flows. In: New Results in Numerical and Experimental Fluid Mechanics VIII. Springer, pp 583–590. 10.1007/978-3-642-35680-3_69

[31] Lintermann A, Meinke M, Schrȯder W (2011) Investigations of the Inspiration and Heating Capability of the Human Nasal Cavity Based on a Lattice-Boltzmann Method. In: Proceedings of the ECCOMAS Thematic International Conference on Simulation and Modeling of Biological Flows (SIMBIO 2011), Brussels

[32] Lintermann A, Meinke M, Schröder W (2013). Fluid mechanics based classification of the respiratory efficiency of several nasal cavities. Comput Biol Med.

[33] Lintermann A, Meinke M, Schrȯder W (2020) Zonal Flow Solver (ZFS): a highly efficient multi-physics simulation framework. International Journal of Computational Fluid Dynamics, pp 1–28. 10.1080/10618562.2020.1742328

[34] Lintermann A, Schlimpert S, Grimmen J, Günther C, Meinke M, Schröder W (2014). Massively parallel grid generation on hpc systems. Comput Methods Appl Mech Eng.

[35] Lintermann A, Schrȯder W (2017). Simulation of aerosol particle deposition in the upper human tracheobronchial tract. Eur J Mech - B/Fluids.

[36] Lintermann A, Schrȯder W (2019). A Hierarchical Numerical Journey Through the Nasal Cavity: from Nose-Like Models to Real Anatomies. Flow Turbul nce Combust.

[37] Litjens G, Kooi T, Bejnordi BE, Setio AAA, Ciompi F, Ghafoorian M, van der Laak JA, van Ginneken B, Sánchez CI (2017). A survey on deep learning in medical image analysis. Med Image Anal.

[38] Lorensen WE, Cline HE (1987). Marching cubes: A high resolution 3D surface construction algorithm. ACM SIGGRAPH Comput Graph.

[39] Maninis KK, Pont-Tuset J, Arbeláez P, Gool LV (2016) Deep retinal image understanding. In: Ourselin S, Joskowicz J, Sabuncu MR, Unal G, Wells W (eds) Medical Image Computing and Computer-Assisted Intervention (MICCAI). Springer International Publishing, pp 140–148

[40] Peksis K, Unger J, Paulauska S, Emsina A, Blumbergs M, Vogt K, Wernecke KD (2018). Relationships among nasal resistance, age and anthropometric parameters of the nose during growth. Rhinol Online.

[41] Qian Yh (1993). Simulating thermohydrodynamics with lattice BGK models. J Sci Comput.

[42] Rivest R (1992) The MD5 Message-Digest Algorithm. 10.17487/rfc1321

[43] Saint-Venant B, Wantzel L (1839) Mėmoire et expėrience sur l’ėcoulement dėterminė par des diffėrences de pressions considėrables. J l’Ėcole Polytechn H.27:85ff

[44] Scadding G, Hellings P, Alobid I, Bachert C, Fokkens W, Gerth van Wijk R, Gevaert P, Guilemany J, Kalogjera L, Lund V, Mullol J, Passalacqua G, Toskala E, Drunen C (2011). Diagnostic tools in rhinology eaaci position paper. Clin Transl Allergy.

[45] Vogt K, Bachmann-Harildstad G, Wernecke KD, Garyuk O, Lintermann A, Nechyporenko A, Peters F (2018). The new agreement of the international RIGA consensus conference on nasal airway function tests. Rhinology.

[46] Vogt K, Jalowayski AA (2010) 4 - Phase-Rhinomanometry, basics and practice 2010 rhinology supplement (21) 20649107

[47] Vogt K, Wernecke KD, Argale M, Kaulina K (2016) Classification of total nasal obstruction in 10,033 cases by 4-phase –rhinomanometry. Roman J Rhinol 6(23). 10.1515/rjr-2016-0017

[48] Vogt K, Wernecke KD, Behrbohm H, Gubisch W, Argale M (2016). Four-phase rhinomanometry: a multicentric retrospective analysis of 36,563 clinical measurements. Eur Arch Oto-Rhino-Laryngol.

[49] Voulodimos A, Doulamis N, Doulamis A, Protopapadakis E (2018). Deep Learning for Computer Vision: A Brief Review. Comput Intell Neurosci.

[50] Waldmann M, Lintermann A, Choi YJ, Schröder W (2020) Analysis of the Effects of MARME Treatment on Respiratory Flow Using the Lattice-Boltzmann Method. In: New Results in Numerical and Experimental Fluid Mechanics XII. Springer, Darmstadt, pp 853–863. 10.1007/978-3-030-25253-3_80

[51] Wilcox DC (1998) Turbulence Modeling for CFD, 2nd editio edn. DCW Industries, La Canada

[52] Xie S, Tu Z (2015) Holistically-nested edge detection. In: Proceedings of the IEEE international conference on computer vision, pp 1395–1403

[53] Youlten L (1980) The peak nasal inspiratory flow meter: a newinstrument for the assessment of the response to immunotherapy in seasonal allergic rhinitis. Allergol Immunopathol 8(344)

